# UBE2S promotes the development of ovarian cancer by promoting PI3K/AKT/mTOR signaling pathway to regulate cell cycle and apoptosis

**DOI:** 10.1186/s10020-022-00489-2

**Published:** 2022-06-03

**Authors:** Mengjun Zhang, Yuan Liu, Yue Yin, Zhenxing Sun, Yan Wang, Zexue Zhang, Fei Li, Xiuwei Chen

**Affiliations:** Department of Gynecology, Harbin Medical University Cancer Hospital, 6 Baojian Rd, Harbin, 150040 China

**Keywords:** Ovarian cancer, Ubiquitin-conjugating enzyme E2S, Prognosis, Malignant biological behavior, Cell cycle, Apoptosis, PI3K/AKT/mTOR signaling pathway

## Abstract

**Background:**

Ovarian cancer is one of the important factors that seriously threaten women's health and its morbidity and mortality ranks eighth among female cancers in the world. It is critical to identify potential and promising biomarkers for prognostic evaluation and molecular therapy of OV. Ubiquitin-conjugating enzyme E2S (UBE2S), a potential oncogene, regulates the malignant progression of various tumors; however, its role in OV is still unclear.

**Methods:**

The expression and prognostic significance of UBE2S at the pan-cancer level were investigated through high-throughput gene expression analysis and clinical prognostic data from TCGA, GEPIA, and GEO databases. 181 patients with OV were included in this study. Cell culture and cell transfection were performed on OV cell lines (SKOV3 and A2780) and a normal ovarian cell line (IOSE80). The expression level and prognostic significance of UBE2S in OV were verified by western blot, immunohistochemistry, and Kaplan–Meier survival analysis. Through cell transfection, CCK-8, Ki-67 immunofluorescence, wound healing, Transwell, clonogenic, and flow cytometry assays, the effect and detailed mechanism of UBE2S knockdown on the malignant biological behavior of OV cells were explored.

**Results:**

UBE2S exhibited abnormally high expression at the pan-cancer level. The results of RT-qPCR and Western blotting indicated that UBE2S was significantly overexpressed in ovarian cancer cell lines compared with normal cell lines (P < 0.05). Kaplan–Meier survival analysis and Immunohistochemistry indicated that overexpression of UBE2S was related to poor prognosis of OV (HR > 1, P < 0.05). Results of in vitro experiments indicated that UBE2S gene knockdown might inhibit the proliferation, invasion, and prognosis of OV cells by inhibiting the PI3K/AKT/mTOR signaling pathway, thereby blocking the cell cycle and promoting apoptosis (P < 0.05).

**Conclusion:**

UBE2S is a potential oncogene strongly associated with a poor prognosis of OV patients. Knockdown of UBE2S could block the cell cycle and promote apoptosis by inhibiting the PI3K/AKT/mTOR pathway and ultimately inhibit the proliferation, migration and prognosis of ovarian cancer, which suggested that UBE2S might be used for molecular therapy and prognostic evaluation of ovarian cancer.

**Supplementary Information:**

The online version contains supplementary material available at 10.1186/s10020-022-00489-2.

## Introduction

Gynecological cancers are malignant tumors of the female reproductive system that affect females worldwide, posing a major threat to society. These cancers include ovarian cancer (OV), cervical squamous cell carcinoma and endocervical adenocarcinoma (CESC), uterine corpus endometrial carcinoma (UCEC), vulvar cancer, and vaginal cancer (Jiang et al. 2020). Among them, OV, UCEC, and CESC are associated with the most cancer-related deaths in women worldwide, with an overall incidence of approximately 150,000/year, 11,000/year, and 311,000/year, respectively. Importantly, 70%, 25%, and 24% of patients with OV, UCEC, and CESC, respectively, are diagnosed when the disease is at an advanced stage (Li et al. 2013). Of particular concern is that OV has the highest mortality rate among cancers of the female reproductive system. Despite advances in common treatment strategies such as the combination of surgery and adjuvant radiotherapy, newer and more effective targeted therapies are required for OV. In recent years, immunotherapy has emerged as an effective cancer treatment strategy. But due to the rapid progression of OV, many patients exhibit treatment resistance and cancer recurrence, thereby resulting in poor clinical outcomes (Eisenhauer 2017; Orr and Edwards 2018). Therefore, there is an urgent need to find personalized, precise treatment targets and effective prognostic biomarkers for gynecological cancers, especially OV.

As a post-translational modification of proteins, ubiquitination is jointly regulated by ubiquitin-like modifier activating enzyme 1 (UBE1), ubiquitin-conjugating enzyme E2 (UBE2), and ubiquitin-protein ligase E3 (UBE3) (Mansour 2018; Popovic et al. 2014). These proteins regulate multiple biological processes such as cell proliferation, differentiation, and migration (Hormaechea-Agulla et al. 2018; Popovic et al. 2014). Mutations in genes or proteins associated with the ubiquitination pathway or alterations in the function of the ubiquitin system are associated with a variety of different human diseases, such as various cancers, neurodegeneration, and metabolic disorders (Choo and Zhang 2009; de Almagro et al. 2017). Ubiquitin-conjugating enzyme E2S (UBE2S) is one of more than 40 ubiquitin-conjugating enzymes E2 that play a vital role in cellular processes (Wu et al. 2010). UBE2S has been reported to be overexpressed in tumors of the gastrointestinal system, urinary system, nervous system, and female reproductive system (Dong et al. 2018; Hu et al. 2017; Lin et al. 2019b; Roos et al. 2011). Additionally, UBE2S drives the proliferation, migration, and invasion of breast, glioma, liver, and other malignant tumor cells and is associated with poor prognosis (Ayesha et al. 2016). Moreover, studies have shown that UBE2S may be related to the chemoresistance of glioblastoma (Hu et al. 2017). Furthermore, studies on the carcinogenic mechanism of UBE2S have shown that its overexpression can promote malignant behaviors of liver cancer cells such as migration and proliferation through the downregulation of p53 (Liu et al. 2017). UBE2S can also promote the occurrence of colorectal cancer by directly interacting with β-catenin (Li et al. 2018). Even in female reproductive system cancers, UBE2S promotes the progression of UCEC by regulating the SOX6/β-catenin signaling pathsway (Lin et al. 2019b). However, the clinical significance, biological function, and molecular mechanism of UBE2S in OV were still obscure.

In this study, the relationship between UBE2S and gynecological cancer, especially OV, was investigated using multi-source high-throughput data analysis. The expression of UBE2S at the mRNA and protein levels was analyzed in three major female reproductive system cancers, and the clinical significance and prognostic value of UBE2S were also evaluated. Subsequently, the expression level of UBE2S in clinical samples and its prognostic evaluation value in OV were verified via a series of in vitro experiments. Finally, the effect of UBE2S on the malignant phenotype of OV was confirmed by knocking down UBE2S in OV cell lines. Overall, the results of multiple high-throughput data, clinical analyses, and a series of rigorous in vitro experiments confirmed that UBE2S might accelerate the cell cycle and inhibit apoptosis by promoting PI3K/AKT/mTOR and ultimately drive the malignant biological behavior of OV cells. Therefore, UBE2S could be a promising target for OV treatment and can be used for prognostic evaluation of patients with OV.

## Materials and methods

### Data collection

The massive transcriptome data of TCGA (The Cancer Genome Atlas, https://portal.gdc.cancer.gov/) and GEPIA (The Gene Expression Profiling Interactive Analysis, http://gepia.cancer-pku.cn) databases provide great convenience for tumor research (Blum et al. 2018; Donehower et al. 2019; Zacchi et al. 2021). The transcriptome data of a variety of cancers were used to analyze the differential expression of UBE2S at the pan-cancer levels from TCGA database and GEPIA database. And the clinical prognostic data of UCEC, OV and CESC were obtained for subsequent survival analysis.

GEO (Gene Expression Omnibus, https://www.ncbi.nlm.nih.gov) database is a widely recognized comprehensive gene expression database (Clough and Barrett 2016). The transcriptome data and clinical prognostic data of OV in 2 datasets were obtained from GEO database for subsequent survival analysis, including GSE18520 and GSE63885. Moreover, 6 datasets containing OV transcriptome data were obtained from the GEO database, including GSE14407 (Tumor = 12, Normal = 12), GSE18520 (Tumor = 53, Normal = 10), GSE40595 (Tumor = 63, Normal = 14), GSE26712 (Tumor = 185, Normal = 10), GSE38666 (Tumor = 25, Normal = 20), and GSE54388 (Tumor = 16, Normal = 6).

The Human Protein Atlas (http://www.proteinatlas.org) database is a public and open platform with a large number of protein expression and immunohistochemistry results (Digre and Lindskog 2021). The protein expression level of UBE2S at the pan-cancer level was obtained based on this database. In addition, the immunohistochemical results of UBE2S in various tissues were obtained, including normal cervical tissue (ID: 3364), ovarian tissue (ID: 4030), endometrial tissue (ID: 1792), and cervical cancer tissue (ID: 2841), ovarian cancer tissue (ID: 3146) and endometrial cancer tissue (ID: 1750).

In the ONCOMINE database, the expression of UBE2S at the pan-cancer level was explored, and the genetic alteration and copy number variation of UBE2S in OV were also explored (Rhodes et al. 2004).

### Patient data and clinical tissue samples

One hundred and eighty-one patients with ovarian cancer in Harbin Medical University Cancer Hospital were included in this study from January 2011 to December 2015. The inclusion criteria for study subjects: (1) Pathologically diagnosed as patients with OV; (2) All patients were diagnosed with OV and followed by OV tumor cytology; (3) All patients received standard adjuvant treatment according to the doctor's recommendation after surgery. The exclusion criteria for study subjects: (1) patients with undiagnosed OV by pathology; (2) patients who have undergone radiotherapy or chemotherapy before surgery; (3) patients with incomplete diagnosis of OV. A follow-up of up to 5 years was carried out for the patients to assess the prognosis. This study was reviewed and approved by the Ethics Committee of Harbin Medical University Cancer Hospital. All patients received and signed the written informed consent. All relevant clinical information was documented in Table 1.

**Table 1:** Baseline data sheet of UBE2S in patients with ovarian cancer

Characteristic	UBE2S expression level		P-value
	LOW	HIGH	
Total Number	32	149	
Pathological type, n (%)			0.560
Clear cell	2 (1.1%)	7 (3.9%)	
Endometrial	4 (2.2%)	12 (6.6%)	
Mucus	10 (5.5%)	39 (21.5%)	
Other	2 (1.1%)	4 (2.2%)	
Serous	14 (7.7%)	87 (48.1%)	
FIGO stage, n (%)			** < 0.001**
I–II	20 (11%)	3 (1.7%)	
III–IV	12 (6.6%)	146 (80.7%)	
Histology grade, n (%)			** < 0.001**
G1–G2	25 (13.8%)	28 (15.5%)	
G3	7 (3.9%)	121 (66.9%)	
Ascites, n (%)			0.103
−	22 (12.2%)	76 (42%)	
+	10 (5.5%)	73 (40.3%)	
Lymph node metastasis, n (%)			0.250
−	25 (13.8%)	98 (54.1%)	
+	7 (3.9%)	51 (28.2%)	
Residual lesion size, n (%)			0.598
0	25 (13.8%)	125 (69.1%)	
2	7 (3.9%)	24 (13.3%)	
Age, mean ± SD	50.81 ± 10.28	54.6 ± 8.92	**0.036**
CA125, meidan (IQR)	459.9 (78.52, 1187.25)	659.6 (318.2, 1459)	**0.046**

### Immunohistochemistry (IHC)

For IHC, the tissue sections were embedded in paraffin in accordance with standard procedures. Tissue blocks were cut to 4 μm and then stained with hematoxylin. Tissue sections were then deparaffinized with xylene and rehydrated in alcohol. Afterwards, endogenous peroxidase activity of tissue sections was removed by incubating in 3% hydrogen peroxide for 10 min at 26 °C. Tissue sections were incubated with a UBE2S primary antibody (1:200; Santa Cruz Biotechnology, China) overnight at 4 °C. Then the tissue sections were incubated at 26 °C for 45 min with a horseradish peroxidase-conjugated secondary antibody and 3,3′-diaminobenzidine (DAB). Tissue sections were developed for 10 min by 3,3′-diaminobenzidine tetrahydrochloride (Dako, Hamburg, Germany). Tissue sections were counterstained for 2 min with hematoxylin. Tissue sections were dehydrated for 8–10 min by being soaked in 100% ethanol. The moisture of the tissue sections was then removed by drying.

Next, the intensity of staining and the percentage of stained tumor cells were recorded under the microscope. The scoring procedure was performed twice independently by two experienced pathologists in a blinded fashion. The intensity of staining was graded and recorded as: 1 (no staining); 2 (weak staining); 3 (moderate staining); 4 (strong staining). Meanwhile, the percentage of stained tumor cells was graded and recorded as: 1 (< 5% positive cells); 2 (5–25% positive cells); 3 (253–50% positive cells); 4 (50–75% positive cells); and 5 (> 75% positive cells). Semi-quantitative analysis of UBE2S protein expression level was performed based on the combined score of the intensity of staining and percentage of stained tumor cells, which ranged from 2 to 9. A combined score greater than 5 was defined as high UBE2S expression, and a combined score less than or equal to 5 was defined as low UBE2S expression. Based on this, 181 patients with OV were assigned to the UBE2S high expression group or UBE2S low expression group and combined with survival data for further survival analysis.

### Cell culture and cell transfection

Human-derived ovarian cancer cell lines (SKOV3, A2780, OVCAR4) and human-derived normal ovarian cells (IOSE80) were purchased from the corresponding institutions (The Cell Bank of the Chinese Academy of Sciences, China). Complete medium was prepared by adding 10% fetal bovine serum (Gibco, Shanghai, China) and 1% penicillin–streptomycin to minimum essential medium (Corning, Shanghai, China). Corresponding cell lines were cultured in this complete medium at 37 °C and 5% CO_2_. The cells were subcultured for subsequent western blotting, cell transfection and a series of further experiments.

The A2780 and SKOV3 cell lines were cultured in a 6-well plate to 50–70% confluence. Cell transfection was performed following standard procedures of cell transfection kit (GenePharma, Shanghai, China). ShRNA-UBE2S was packaged in lentiviral vector by the corresponding institution GenePharma, Shanghai, China). Cell transfection was performed at a cell density of 2 × 10^5^/well. The original medium was replaced with 2 mL of fresh medium containing 6 μg/mL polybrene. An appropriate amount of suspension of lentivirus loaded with sh-UBE2S was added. Incubate at 37 °C. Culture was continued for 24 h, replacing the lentivirus-containing medium with fresh medium. Next, the cells were subcultured. Stably transfected cells were selected by puromycin. The corresponding lentivirus was marked with a GFP tag. Successfully transfected cells showed green fluorescence under a fluorescence microscope. The transfection efficiency was verified by immunofluorescence and western blotting, and then a series of further experiments were carried out.

### Reverse transcription quantitative polymerase chain reaction (RT-qPCR)

RT-qPCR was conducted for investigating the expression level of UBE2S in ovarian cancer cell lines and normal cell lines. Total RNA of corresponding cell lines was isolated by TRIzol (Thermo Fisher Scientific, Waltham, USA). Then, the solubility of total RNA was measured with a NanoDrop One spectrophotometer (Thermo Fisher Scientific, Waltham, USA). After that, the cDNA was obtained by reverse transcription. At last, RT-qPCR was conducted for investigating the expression of UBE2S with NovoStart SYBR qPCR SuperMix Plus (Novoprotein, Shanghai, China). GAPDH was set as an internal reference and its primer sequences were as follows: (GAPDH-F:5′-CAAGGTCATCCATGACAACTTTG-3′, GAPDH-R:5′-GTCCACCACCCTGTTGCTGTAG-3′). The primer sequences of UBE2S were as follows: (UBE2S-F:5′-CGATGGCATCAAGGTCTTTCCC-3′, UBE2S-R:5′-CAGCAGGAGTTTCATGCGGAAC-3′). Thermal cycling conditions were set as follows: the first step was pre-denaturation at 95 °C for 3 min, and the second step was denaturation at 95 °C for 10 s, and the third step was annealing and extension at 60 °C for 30 s, and finally the second and third steps were performed and repeated for 40 cycles. Finally, the relative expression of UBE2S was calculated by the 2^−ΔΔCT^ method.

### Western blotting

Western blot was first used to determine the expression level of UBE2S in ovarian cancer cell lines and normal cell lines, then used to determine the transfection efficiency after cell transfection, and finally used to determine the expression levels of cell cycle, apoptosis and PI3K/AKT/mTOR signaling pathway-related proteins after cell transfection. The corresponding antibody summary table is shown in Additional file 3: Table S3. Corresponding cell lines were lysed in lysis buffer supplemented with 1% protease inhibitors. Cell lysates were centrifuged at 12,000×*g* for 15 min at 4 °C, immediately after which the supernatant was collected. The corresponding protein concentrations were quantified by using the Bradford kit (Thermo Fisher Scientific, Waltham, USA). Proteins of different molecular weights were separated by electrophoresis in 10% SDS-PAGE gels. The proteins in the gel were then transferred to methanol-treated polyvinylidene fluoride (PVDF) membranes. PVDF membranes were transferred to a shaker and blocked for 2 h with 5% nonfat dry milk in TBS-T. The anti-UBE2S primary antibody and PVDF membrane were mixed and placed in an incubation box for overnight incubation at 4 °C. The PVDF membrane was washed with PBS. The PVDF membrane and horseradish peroxidase-labeled rabbit secondary antibody were mixed and placed in an incubation box for 1 h at 26 °C. After that, PBS was again used to wash the PVDF membrane. Finally, the western blot on this PVDF membrane was developed by using ECL developer, and imaging was performed by using a charge-coupled camera LAS4000 (Fujifilm, Tokyo, Japan). The grayscale values of the bands were measured by ImageJ software (version v.1.52). The relative expression levels of proteins were represented by the grayscale values of the bands.

### Cell proliferation assay based on CCK8 assay

The cell proliferation ability after cell transfection was investigated by CCK8 (Cell Counting Kit-8). Collect and resuspend the transfected cells in the logarithmic growth phase. The concentration of the cell suspension was diluted to 2 × 10^4^ cells/mL. The corresponding cell suspensions were evenly distributed in 96-well plates in a volume of 100 μL per well. 10 μL of CCK-8 reagent (Yeasen, Shanghai, China) was added added to the medium. Cells were cultured in a suitable environment and checked for cell viability at 0, 24, 48 and 72 h. Cells were cultured for 2 h at 37 °C in a 5% CO_2_ incubator. A microplate reader was used (Thermo Fisher Scientific, Waltham, USA) to measure OD (optical density) at a wavelength of 450 nm.

### Ki-67 cell immunofluorescence

Select transfected cells with a cell density of about 50%-65% to make cell slides. Cells were fixed by 1 mL of 4% paraformaldehyde. 0.5% Triton X-100 was used for cell penetration at 26 °C for 20 min. Wash with PBS and block with 10% goat serum at room temperature for 2 h. Add sufficient primary antibody (Ki-67, 1:100) to each well at the appropriate concentration. Incubate overnight in a humidified cabinet at 4 °C. Then add the secondary antibody and incubate at 37 °C in the dark at room temperature for 1 h. Add DAPI dropwise to the glass slide. Incubate in the dark for 10 min. Use mounting fluid to mount the slide. Then observe and collect images under a fluorescence microscope. KI67-positive fluorescent cells were considered proliferating cells. The numbers of living and proliferating cells were measured and recorded by using ImageJ software (version v.1.52).

### Clonogenic assay

Transfected cells were digested, resuspended and evenly seeded in 6-well plates in appropriate numbers (300 cells/well). The cells in the 6-well plate were then cultured using complete medium. Colony formation was observed after ten days. Cell colonies were fixed at 26 °C for 30 min by using 4% paraformaldehyde. Cell colonies were stained for 5 min by using 1% crystal violet. The colonies formed were observed and imaged under the microscope. The number of colonies formed was measured and counted by ImageJ software (version v.1.52).

### Wound-healing assay

The transfected ovarian cancer cells were seeded in a 6-well plate at an appropriate density. When the cells have grown to 95% confluence, use a 200 μL sterile pipette tip to scribe each hole straight to create a scratch, and then wash with 1 × PBS 3 times to remove the sloughed cells. Change complete medium to fresh serum-free medium. The corresponding cells were placed in a suitable environment for 48 h. Use a microscope to take pictures of the scratches on the same part at 40 times magnification at 24 h and 48 h. Measure the scratch area to calculate the wound-healing rate with ImageJ software (version v.1.52).

### Transwell assay

Transfected ovarian cancer cells were collected and centrifuged. Resuspend it to a specific density (1 × 10^6^ cells/mL) with serum-free medium. Then 100 μL of the transfected ovarian cancer cell suspension was seeded into the upper chamber of Transwell plate (Corning Costar, Shanghai, China). 600 µL of minimum essential medium and 20% FBS were added to the lower chamber of the Transwell plate. Transwell plates were incubated for 24 h at 37 °C in 5% CO_2_. Cells remaining in the upper chamber were removed by using cotton swabs. Then cells that crossed the membrane into the lower compartment were fixed in 95% ethanol for 15 min. The above cells were stained for 15 min by using 0.1% crystal violet. PBS was then used to remove excess crystal violet stain. A microscope was used to observe the cells and take pictures. The number of migrating cells was measured and recorded with ImageJ software (version v.1.52).

### Flow cytometry related to cell cycle and apoptosis

Transfected cells were digested, centrifuged and resuspended. Pre-chilled 75% ethanol was added to the cell suspension. Afterwards, the test tube containing the cell suspension was placed at − 20 °C overnight. 10 μL of RNase solution (Yeasen, Shanghai, China) was used to remove RNA impurities from the cell suspension. 10 μL of propidium iodide (PI) was added to the cell suspension for staining for 20 min. The distributions of cell cycle and apoptosis were detected by flow cytometry (Canto plus, BD, USA).

### UBE2S-related gene enrichment analysis

UBE2S-related gene enrichment analysis was conducted for investigating the promising molecular mechanism of UBE2S in OV. Go (Gene Ontology) analysis and KEGG (Kyoto Encyclopedia of Genes and Genomes) analysis were conducted by David tools (Dennis et al. 2003; Gaudet et al. 2017; Kanehisa and Goto 2000). Pearson analysis was performed to investigate the list of genes positively or negatively associated with UBE2S. Use the "tidyr" and "ggplot2" R packages for visualization. In addition, the annotations of the potential pathways were recorded.

### Statistical analysis

R software (version v.3.6.1) and GraphPad Prism (version v.9.0) were used for statistical analysis. ImageJ software (version v.1.52) was used to measure various parameters under the microscope. Student's t-test, Kruskal Wallis test, and Wilcoxon signed-rank test was widely used to compare statistical indicators. Kaplan–Meier survival analysis was conducted to investigate the correlation of UBE2S with prognosis in ovarian cancer patients. The receiver operating characteristic (ROV) curve was performed to investigate the prognostic value of UBE2S. Statistical significance for this study was defined as P < 0.05.

## Results

### Expression and prognostic significance of UBE2S in pan-gynecological cancer

**Fig. 1:**
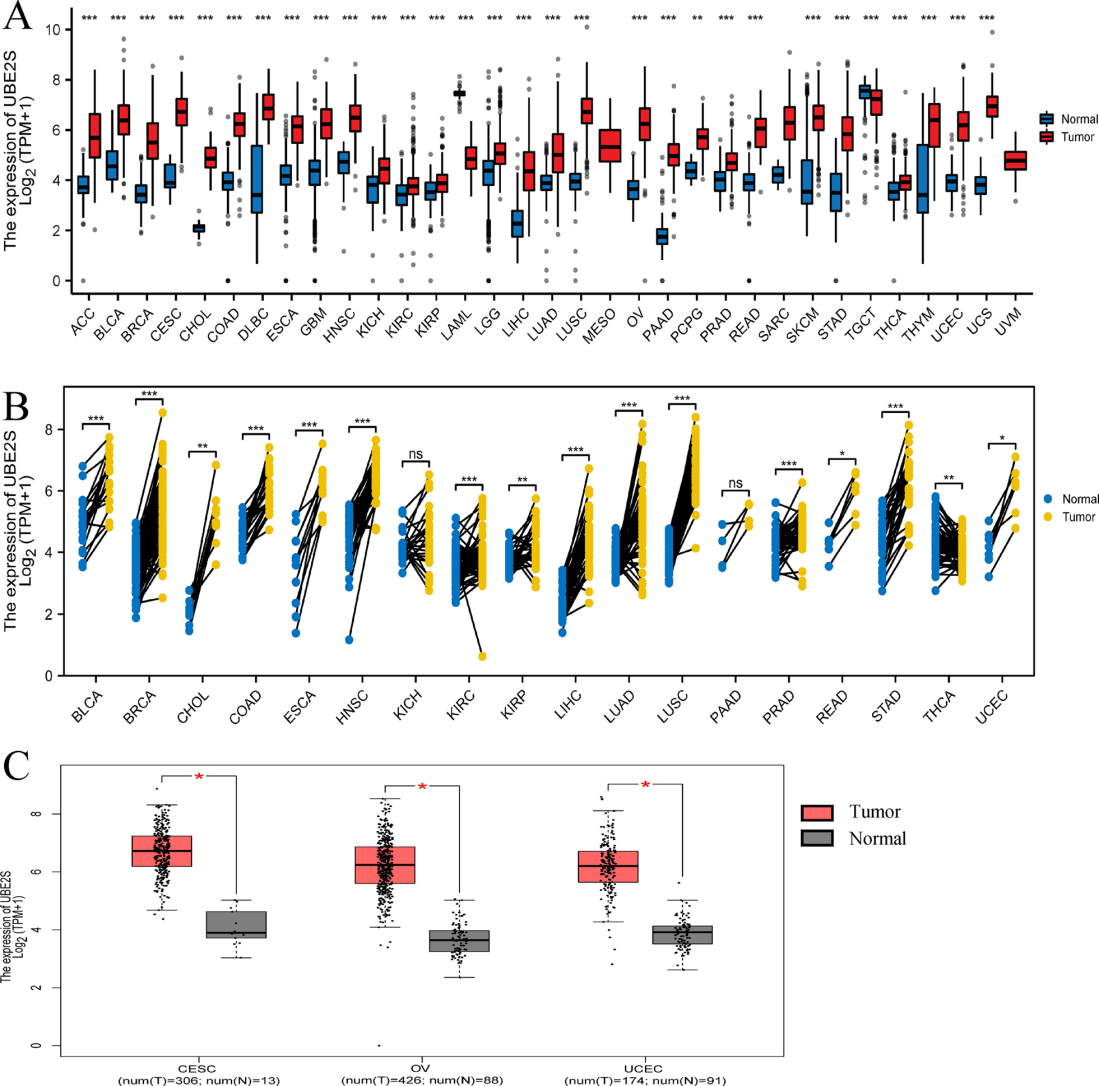
UBE2S mRNA expression at the pan-cancer level. **A** UBE2S mRNA expression level in 33 types of human tumor tissues and corresponding normal tissues from GEPIA databases. UBE2S was significantly highly expressed in 18 types of cancer tissues. **B** UBE2S mRNA expression level in 18 types of human tumor tissues and matched adjacent normal tissues from TCGA databases. UBE2S was significantly highly expressed in 15 types of cancer tissues. **C** UBE2S mRNA expression level in CESC, OV, UCEC tissues and the corresponding normal control tissues (normal cervix, normal ovary and normal endometrial tissue) from GEPIA databases. The number of tissue samples was detailed in parentheses in the figure. (*P < 0.05, **P < 0.01, ***P < 0.001, ****P < 0.0001.)

First, the expression level of UBE2S was analyzed at a pan-cancer level. Gene expression analysis on 33 types of human tumor tissues and corresponding normal tissues indicated that UBE2S expressed higher in 18 types of cancer tissues based on the GEPIA database (Fig. 1A and Additional file 1: Table S1, Additional file 2: Table S2). Furthermore, gene expression analysis on 18 types of human tumor tissues and matching adjacent normal tissues revealed that UBE2S expressed higher in 15 types of cancer tissues based on the TCGA database (Fig. 1B). These results indicate that UBE2S is a potential broad-spectrum oncogene involved in varieties of human cancers.

**Fig. 2:**
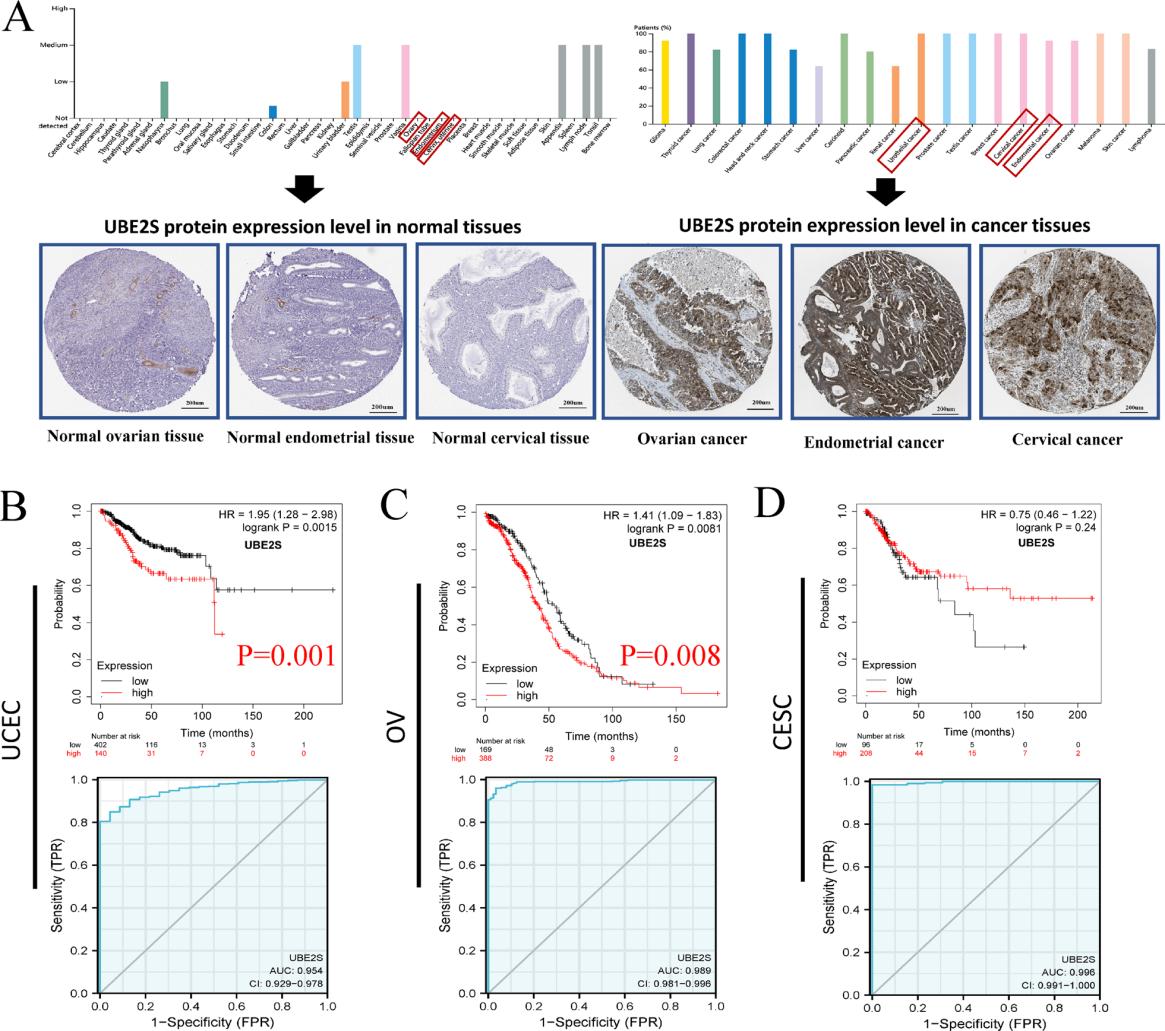
UBE2S protein expression and prognosis analysis in major gynecological cancer tissues. **A** Immunohistochemical staining. The staining intensity represents the protein expression level of UBE2S in 45 types of normal organs and 20 types of cancer tissues in the HPA database. UBE2S was low expressed in normal ovarian tissue, cervical tissue and endometrial tissue. UBE2S was overexpressed in ovarian cancer tissues, cervical cancer tissues and endometrial cancer tissues. **B–D** Survival curve and ROC curve of UBE2S high/low expression group in UCEC, OV and CESC. UCEC (HR 1.95, P = 0.0015, AUC = 0.954), OV (HR 1.41, P = 0.0081, AUC = 0.989) and CESC (HR 0.75, P = 0.24, AUC = 0.996)

Importantly, in the GEPIA database, the mRNA expression of UBE2S in CESC, OC, and UCEC tissues was higher than that in corresponding normal tissues (normal cervix, normal ovary, and normal endometrial tissues) (Fig. 1C). Furthermore, in the HPA database, the protein expression of UBE2S in CESC, OC, and UCEC tissues was higher than the protein expression of UBE2S in corresponding normal tissues (normal cervix, normal ovary, and normal endometrial tissues) (Fig. 2A). The results of gene and protein expression analysis strongly confirmed that UBE2S is a potential broad-spectrum oncogene in major gynecological malignancies.

Based on the abnormally high expression of UBE2S in pan-gynecological cancers, the Kaplan–Meier method was performed to explore the overall survival (OS) of patients with gynecological cancer and correlate it with the prognosis of patients (Fig. 2B–D). The ROC curve was conducted for investigating the prognostic value of UBE2S in gynecological cancers. Compared with that of the UBE2S high expression group, the OS of the UBE2S low expression group was longer in patients with UCEC (P = 0.0015) and OC (P < 0.0081). Moreover, the level of UBE2S expression has a strong prognostic assessment value for patients with UCEC (AUC = 0.954) and OC (AUC = 0.989).

### Expression and prognostic significance of UBE2S in OV

**Fig. 3:**
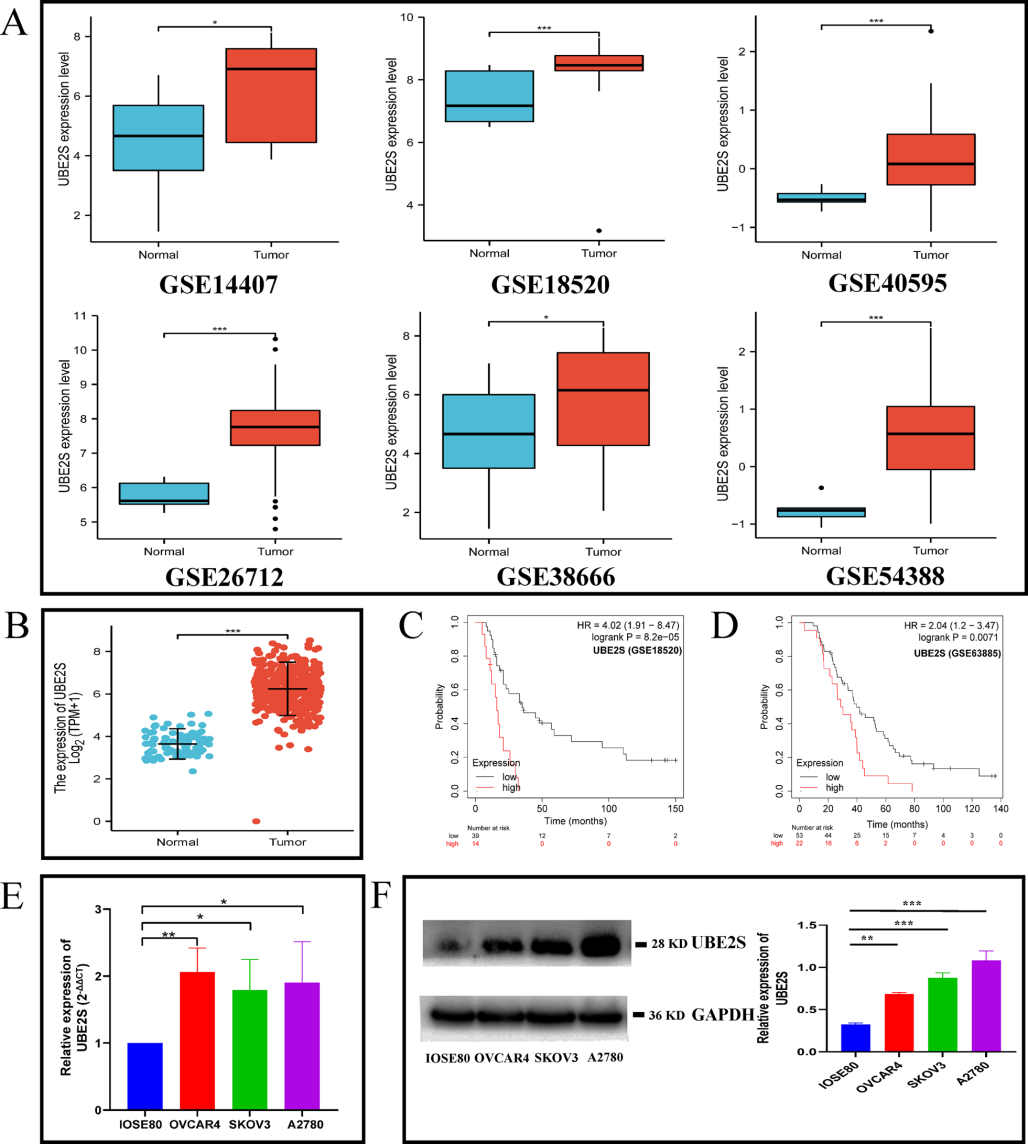
The expression of UBE2S in OV according to high-throughput database and low-throughput in vitro experiments. **A** The mRNA expression level of UBE2S in ovarian cancer tissues and corresponding control tissues based on six GEO database (GSE14407, GSE18520, GSE40595, GSE26712, GSE38666 and GSE54388). **B** Scatter plot of UBE2S expression levels in ovarian cancer tissues and corresponding control according to TCGA data (tumor = 427, normal = 88). **C** Survival curve of UBE2S high/low expression group in ovarian cancer GEO dataset GSE18520 (HR 4.02, P = 0.000082). **D** Survival curve of UBE2S high/low expression group in ovarian cancer GEO dataset GSE63885 (HR 2.04, P = 0.0071). **E** The mRNA expression levels of UBE2S in OV cell lines (SKOV3, A2780, OVCAR4) and normal cell lines (IOSE80) based on RT-qPCR. **F** The protein expression levels of UBE2S in ovarian cancer cell lines (A2780, SKOV3, OVCAR4) and a normal ovarian cell line (IOSE80) based on western blotting in this study

Next, the expression level and clinical significance of UBE2S in OV specifically were explored. At the pan-cancer level, UBE2S showed an abnormally high expression and considerable genetic alteration and copy number variation, especially in OV (Additional file 4: Fig. S1). Analysis of six OV-related datasets of the GEO database and one OV-related datasets of the TCGA database also indicated the abnormally overexpression of UBE2S in OV tissues compared to corresponding control tissues (Fig. 3A, B). Besides, two datasets (GSE18520 and GSE63885) containing gene expression and prognostic data indicated that the OS of the UBE2S high expression group was shorter compared to the UBE2S low expression group (P < 0.05, HR > 1) (Fig. 3C, D). Therefore, analysis using different public databases confirmed that the overexpression of UBE2S in OV could lead to a poor prognosis.

**Fig. 4:**
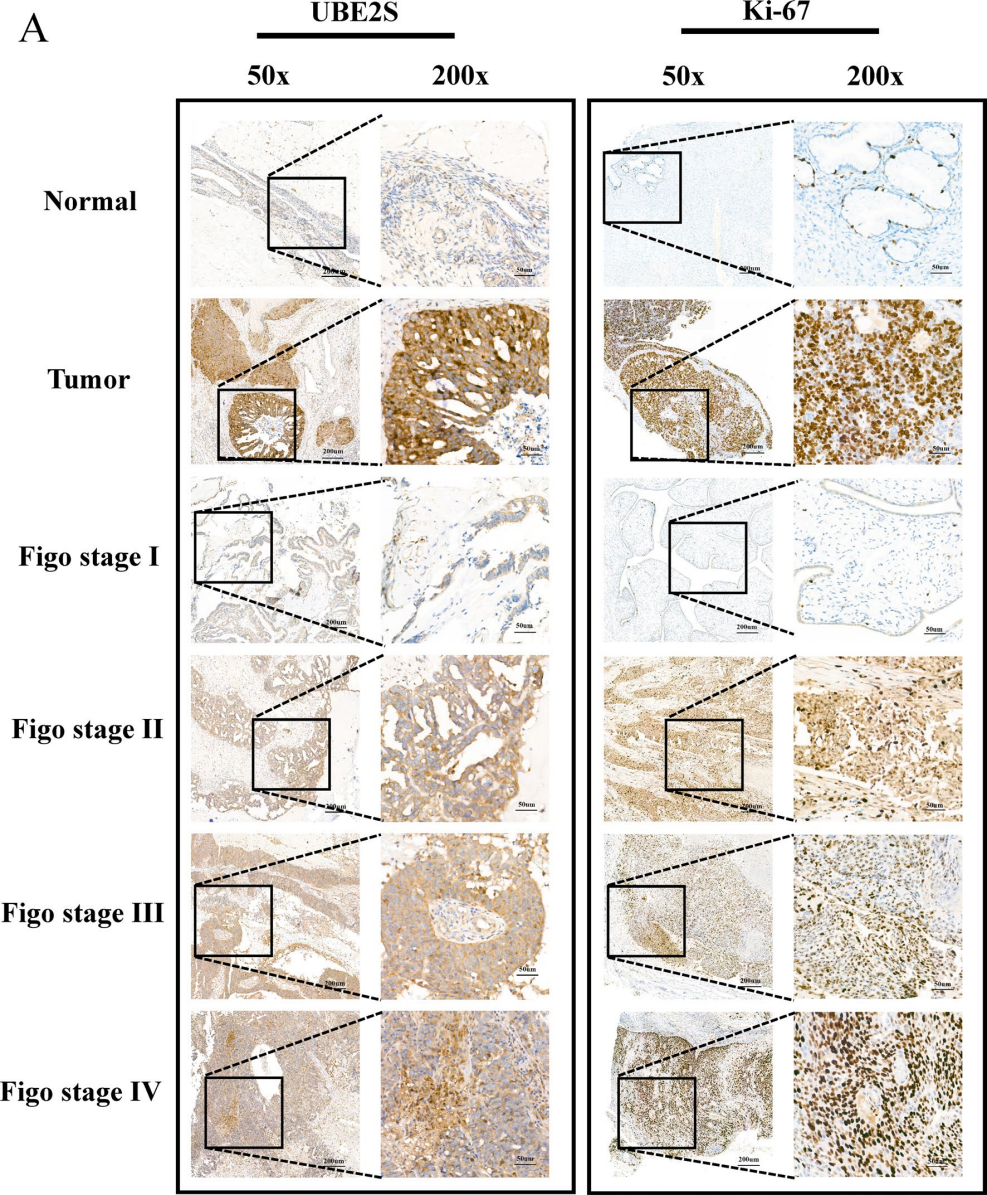
The expression level of UBE2S and Ki67 in OV based on immunohistochemistry in this study. **A** The immunohistochemistry atlas of normal ovarian tissue, ovarian cancer tissue and ovarian cancer tissues of different FIGO stages. × 50 and × 200 (partial enlargement) microscopic views were shown. The intensity of staining represents the level of expression

**Fig. 5:**
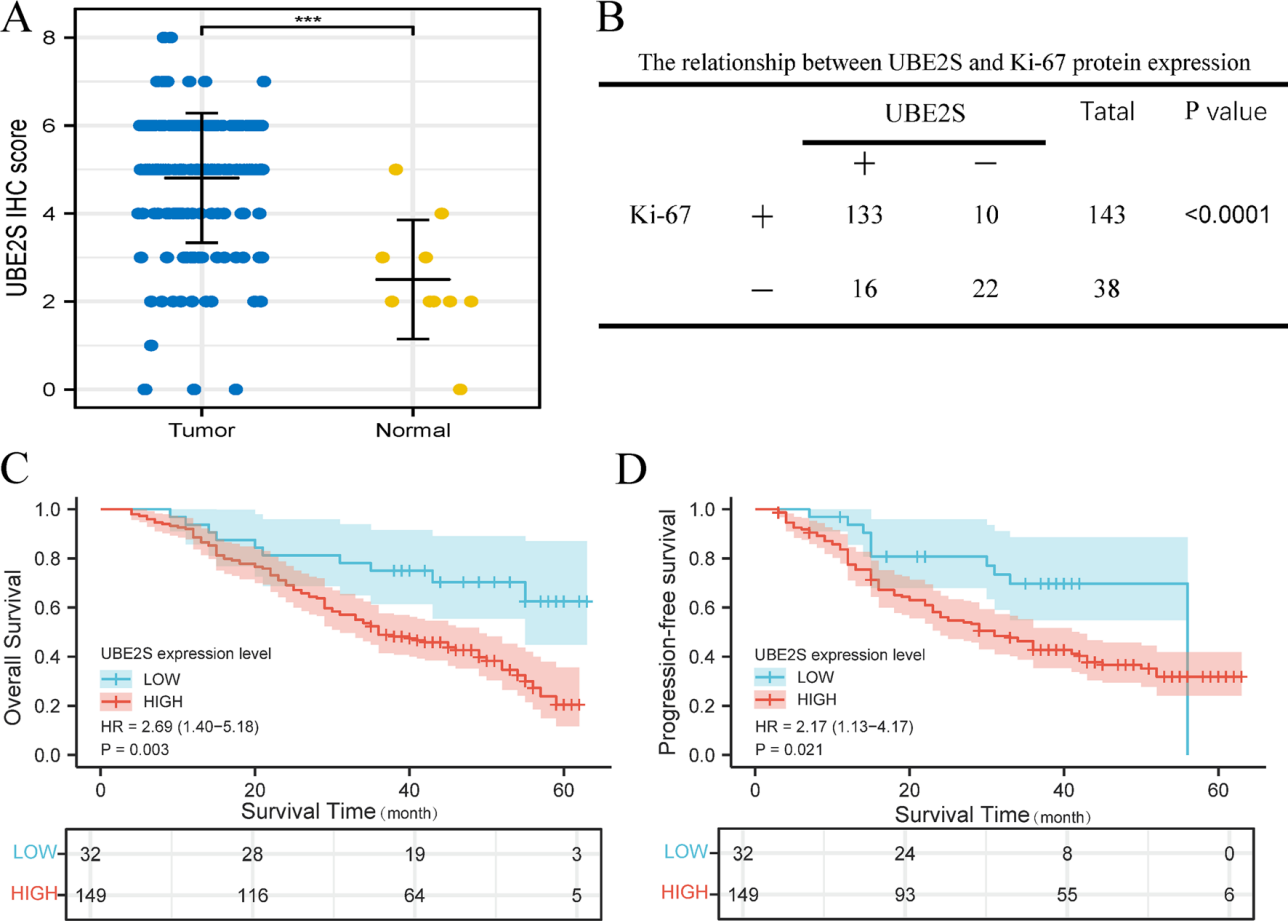
Expression differences in UBE2S protein expression levels, Ki67 correlation and prognostic significance in OV patients. **A** Immunostaining scoring of UBE2S protein in ovarian cancer and normal ovarian tissue. **B** Statistical table of the correlation distribution of UBE2S protein and Ki67 expression in ovarian cancer tissue. **C** The relationship between UBE2S expression levels with OS in OV patients. **D** The relationship between UBE2S expression levels with PFS in OV patients. According to the results of IHC staining, OV patients were assigned to the UBE2S high expression group or UBE2S low expression group. Kaplan–Meier survival analysis was then carried out

Subsequently, in vitro experiments and clinical sample collection were conducted to verify the results of analysis of different public databases. The RT-qPCR results implied that the mRNA expression level of UBE2S in OV cell lines (SKOV3, A2780, and OVCAR4) was significantly higher than that in normal ovarian cells (IOSE80), as shown in Fig. 3E. Results of western blotting in Fig. 3F indicated that the protein expression level of UBE2S in OV cells (SKOV3, A2780, and OVCAR4) was significantly higher than the protein expression level of UBE2S in normal ovarian cells (IOSE80). Furthermore, IHC data on the clinical samples indicated that the protein expression levels of UBE2S and Ki-67 (a characteristic marker of cell proliferation) in OV tissues (based on staining scores) were higher than those in normal ovarian tissues (Figs. 4 and 5A–B). Moreover, with increasing FIGO stage, the protein expression levels of UBE2S and Ki-67 showed an upward trend, thereby suggesting that UBE2S is a potential oncogene in OV associated with regulation of proliferation and malignant progression (Fig. 4). Results of the survival analysis of patients with OV indicated that the OS time and PFS time of OV patients in the UBE2S low expression group were longer than those in the UBE2S high expression group (Fig. 5C, D). Univariate and multivariate analysis identified UBE2S as an independent factor for ovarian cancer (Table 2).

**Table 2:** Univariate and multivariate Cox regression analyses of UBE2S

Characteristics	Total (N)	Univariate analysis		Multivariate analysis	
		Hazard ratio (95% CI)	P value	Hazard ratio (95% CI)	P value
Grade	181				
G1-G2	53				
G3	128	5.786 (3.009–11.128)	** < 0.001**	6.184 (2.862–13.362)	** < 0.001**
FIGO stage	181				
I–II	23				
III–IV	158	3.773 (1.535–9.278)	**0.004**	3.732 (1.154–12.076)	**0.028**
Lymph node metastasis	181				
−	123				
+	58	0.677 (0.442–1.037)	0.073		
Residual lesion size	181				
0	150				
2	31	0.489 (0.267–0.894)	**0.020**	0.541 (0.294–0.995)	**0.048**
UBE2S expression level	181				
High	149				
Low	32	0.372 (0.193–0.717)	**0.003**	2.368 (0.888–6.320)	**0.045**

In summary, results of gene expression analysis, in vitro experiments, and IHC on clinical samples proved that UBE2S shows an abnormally high expression in OV and is associated with poor prognosis of OV patients.

### Verification of knockdown efficiency of UBE2S gene.

After confirming the abnormal overexpression (the genetic level) and prognostic significance (the clinical level) of UBE2S in ovarian cancer, the influence of UBE2S on the biological behavior of cells (the level between the genetic level and the clinical level) was tried to explored.

**Fig. 6:**
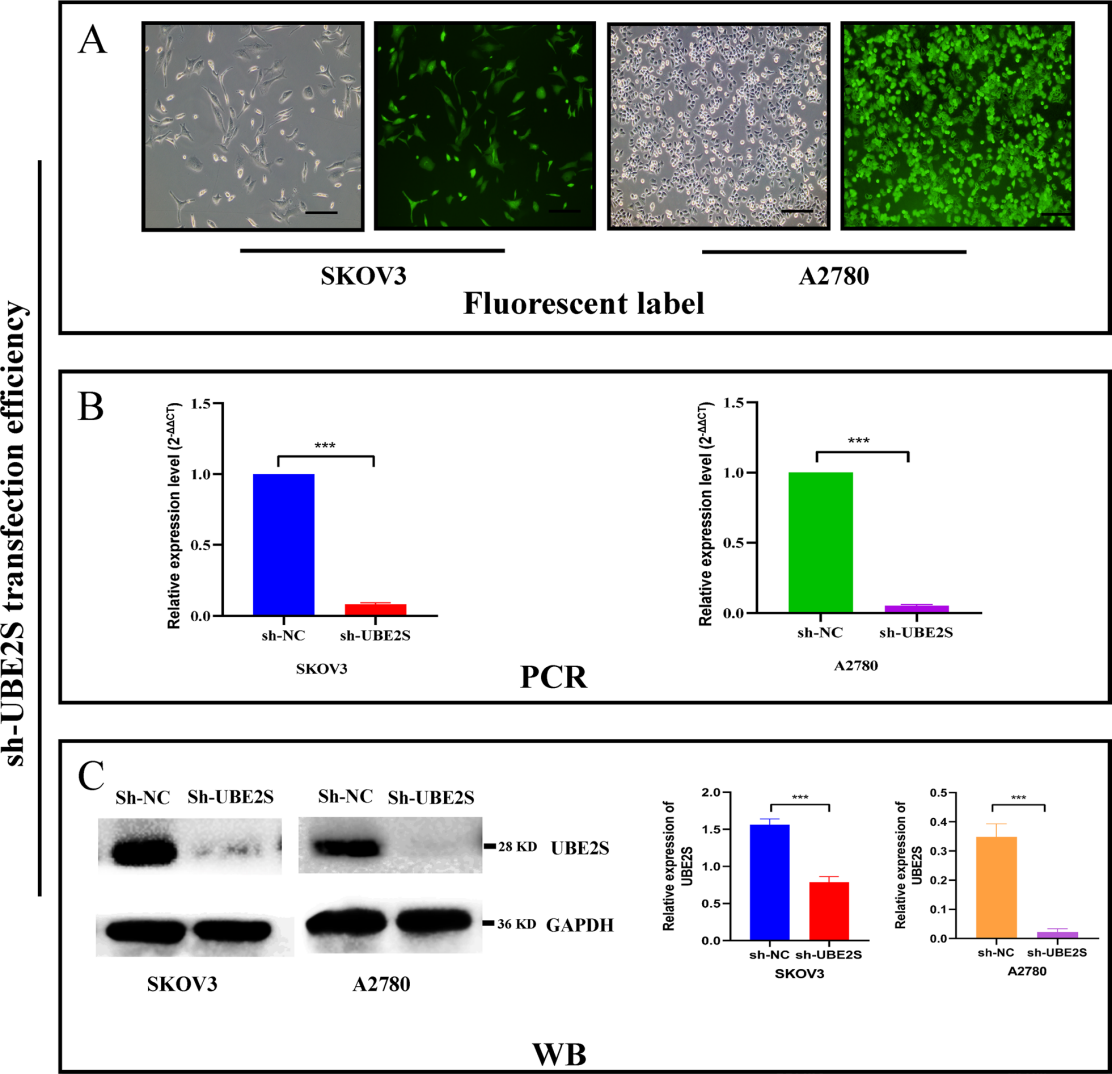
The knockdown efficiency of sh-UBE2S in OV cells base on immunofluorescence and Western blotting. **A** The transfection efficiency of sh-UBE2S evaluated by the non-fluorescence channel and the green fluorescence channel of the fluorescence microscope. The cells under the non-fluorescent channel represent the total live cells. The cells under the green fluorescence channel represent live cells that have been successfully transfected. **B** RT-qPCR after cell transfection. **C** Western blot image and corresponding statistics after cell transfection. (*P < 0.05, **P < 0.01, ***P < 0.001.)

The further research plan was to perform UBE2S gene knockdown in OV cells (A2780 and SKOV3), and then verify the biological behavior of OV cells. As can be seen from Fig. 6A, the proportion of successfully transfected cells (UBE2S gene knockdown cells) was determined based on the fluorescence intensity, which represents a high transfection efficiency. As shown in Fig. 6B, C, results of the PCR and western blotting analyses implied that the expression of UBE2S was higher in the sh-NC group compared to the expression in the sh-UBE2S group, thus confirming an appropriate knockdown efficiency of UBE2S at the protein and mRNA levels.

### The effect of UBE2S knockdown on the malignant biological behavior of ovarian cancer cells

After verifying the knockdown efficiency of UBE2S, a series of in vitro experiments were performed for investigating the effect of UBE2S knockdown on the malignant biological behavior in the OV cells.

**Fig. 7:**
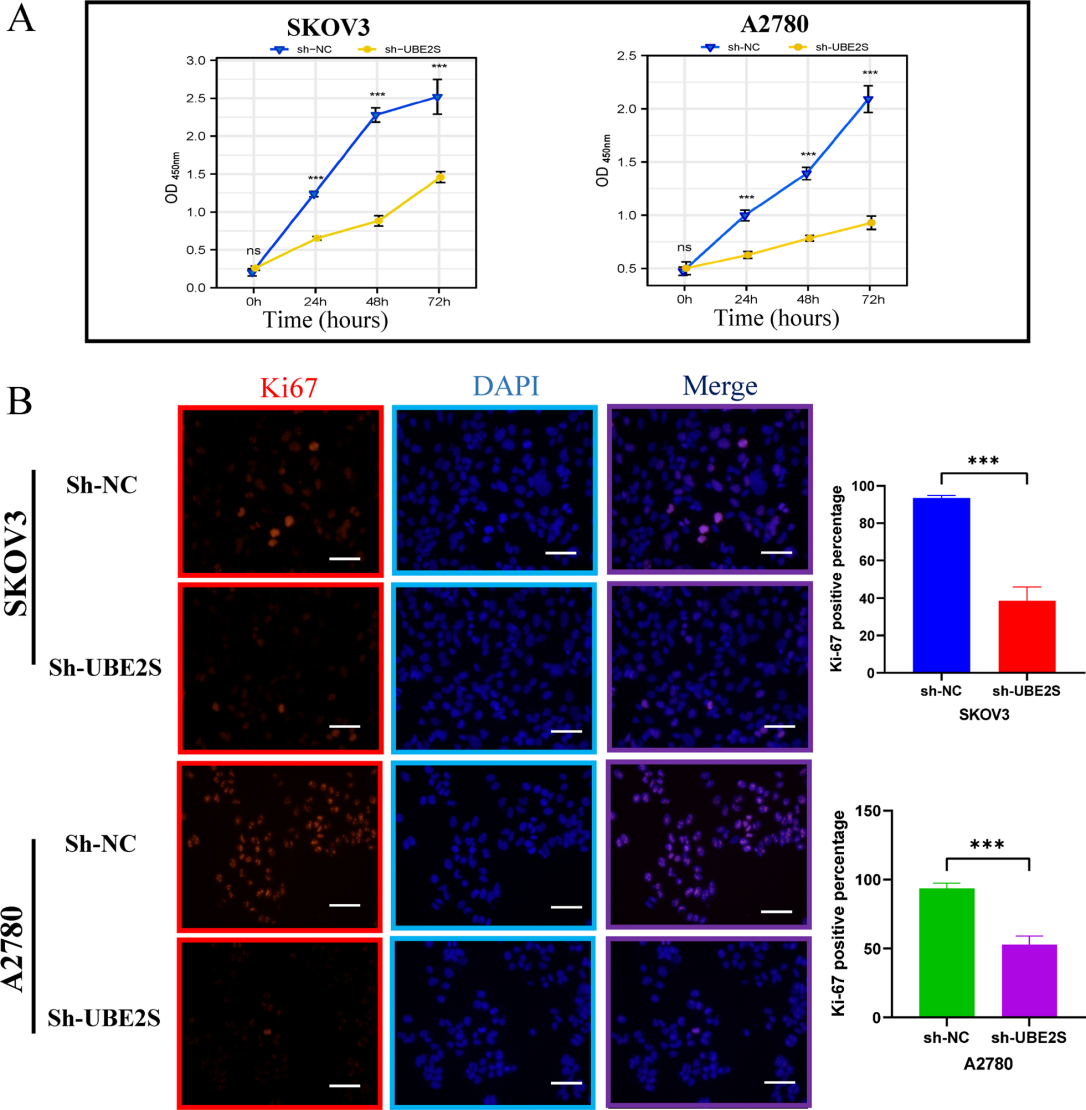
The impact of UBE2S gene knockdown on the proliferation of OV cells. **A** CCK8 experiment results of A2780 and SKOV3 cell lines after cell transfection. The OD values measured at 450 nm wavelength at 0 h, 24 h, 48 h, and 72 h were displayed, which represented the cell proliferation rate. **B** Ki-67 immunofluorescence staining of A2780 and SKOV3 cell lines after cell transfection. Ki67 red staining represents cells in a state of proliferation. The DAPI blue nuclear staining represents total living cells. Merge represents the merging of two images. Related statistical graphs were also displayed

**Fig. 8:**
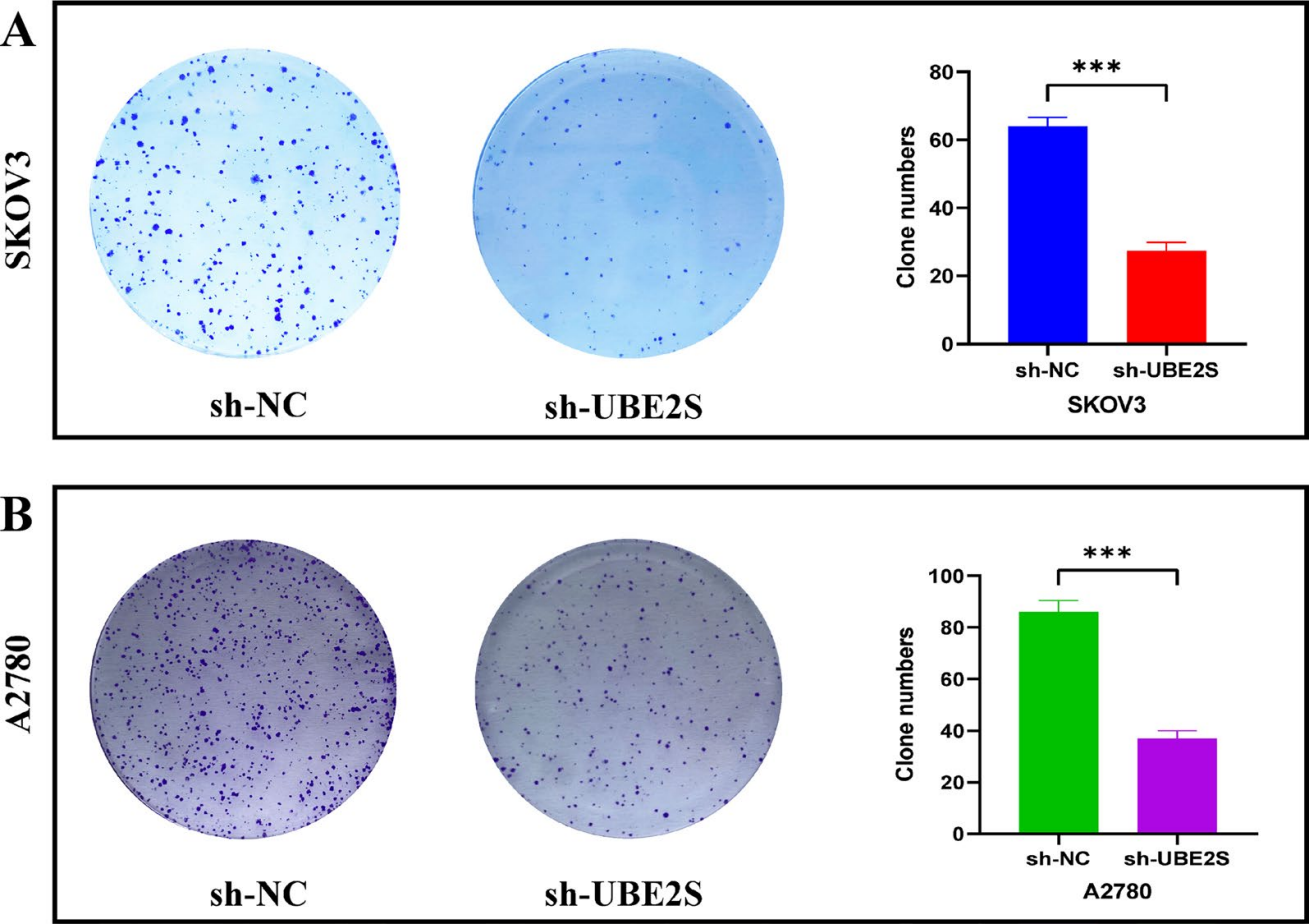
The effect of UBE2S gene knockdown on the clonogenic assay. **A** The results and statistics of cell clone formation assay of SKOV3 cell lines after cell transfection. **B** The results and statistics of cell clone formation assay of A2780 cell lines after cell transfection

First, the CCK8 assay results showed that the optical density in the sh-NC group was higher than the optical density in the sh-UBE2S group at 24 h, 48 h, and 72 h in A2780 and SKOV3 cell lines (Fig. 7A). Thereafter, the results of the Ki-67 immunofluorescence assay indicated that among OV cells, the percentage of Ki-67 fluorescence positive cells was higher in the sh-NC group compared to the percentage in the sh-UBE2S group (Fig. 7B). Interestingly, the results of the clonogenic assay implied that in OV cells, more clones were formed in the sh-NC group than in the sh-UBE2S group at day 10 (Fig. 8A, B). Therefore, these results implied that the proliferation was inhibited after UBE2S knockdown in the OC cells.

**Fig. 9:**
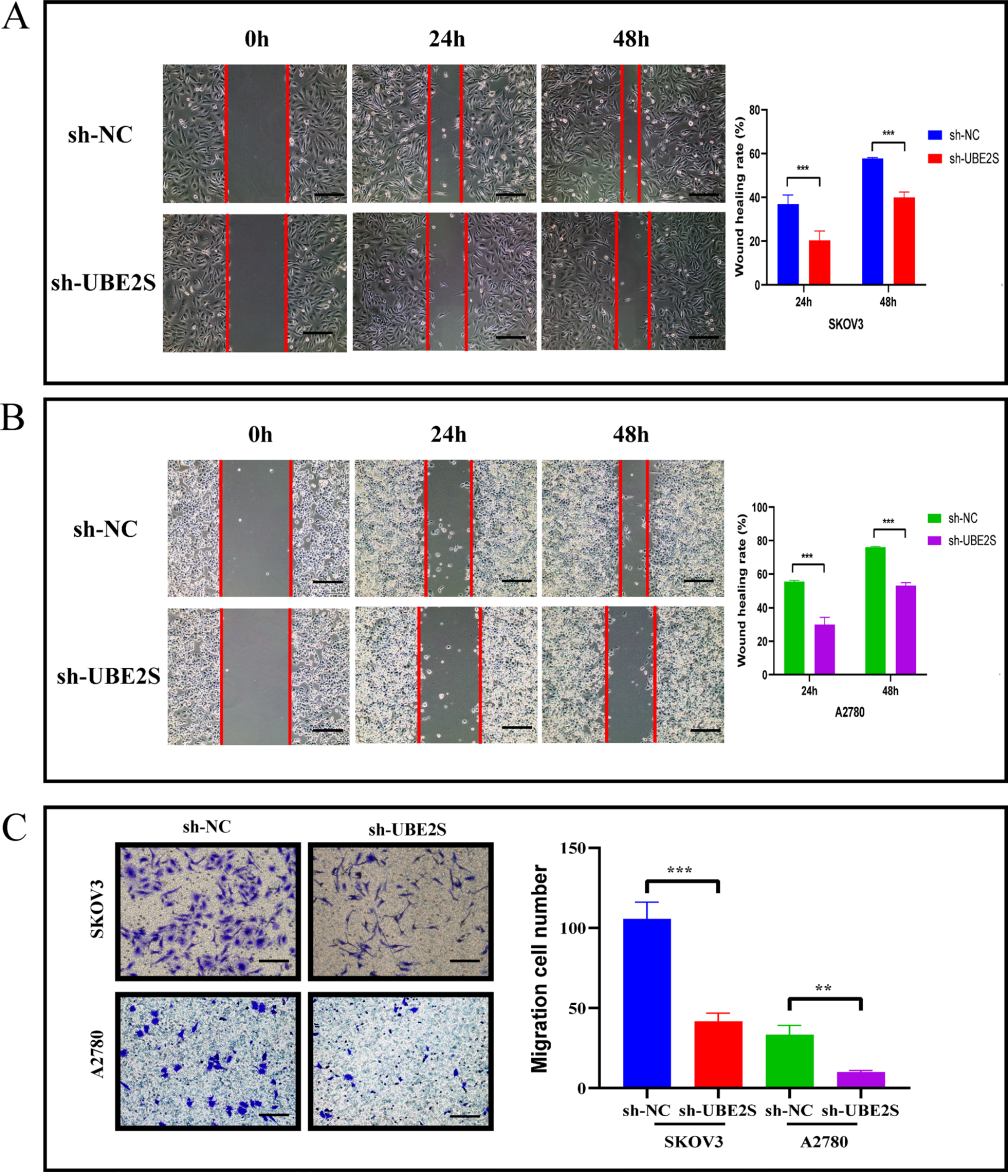
The effect of UBE2S gene knockdown on the migration of OV cells. **A** Wound-healing assay results and statistics of SKOV3 cell line after cell transfection. **B** Wound-healing assay results and statistics of the A2780 cell line after cell transfection. The wound-healing rate was measured at 0, 24, and 48 h, which represented the migration capacity. **C** Transwell assay results and statistics of OV cells after cell transfection. The number of migrating cells was measured at 24 h, which represented the ability to migrate

In contrast, the results of the wound-healing assay indicated that the wound-healing rate of cells in the sh-NC group was significantly higher than that the wound-healing rate in the sh-UBE2S group at 24 and 48 h in OV cells (Fig. 9A, B). Besides, the Transwell assay results suggested that the number of migrating cells in the sh-NC group was more than the number in the sh-UBE2S group in OV cells (Fig. 9C). All of the above results also confirmed that the migration were inhibited after UBE2S knockdown in the OC cells.

Overall, the results of the in vitro experiments strongly suggest that the biological behaviors such as the proliferation and migration were suppressed after UBE2S knockdown in the OC cells.

### Analysis of the mechanism by which UBE2S affects the malignant progression of OV cells

After confirming the effect of UBE2S knockdown on the malignant behavior of OV cells, the specific mechanism of action of UBE2S was analyzed.

**Fig. 10:**
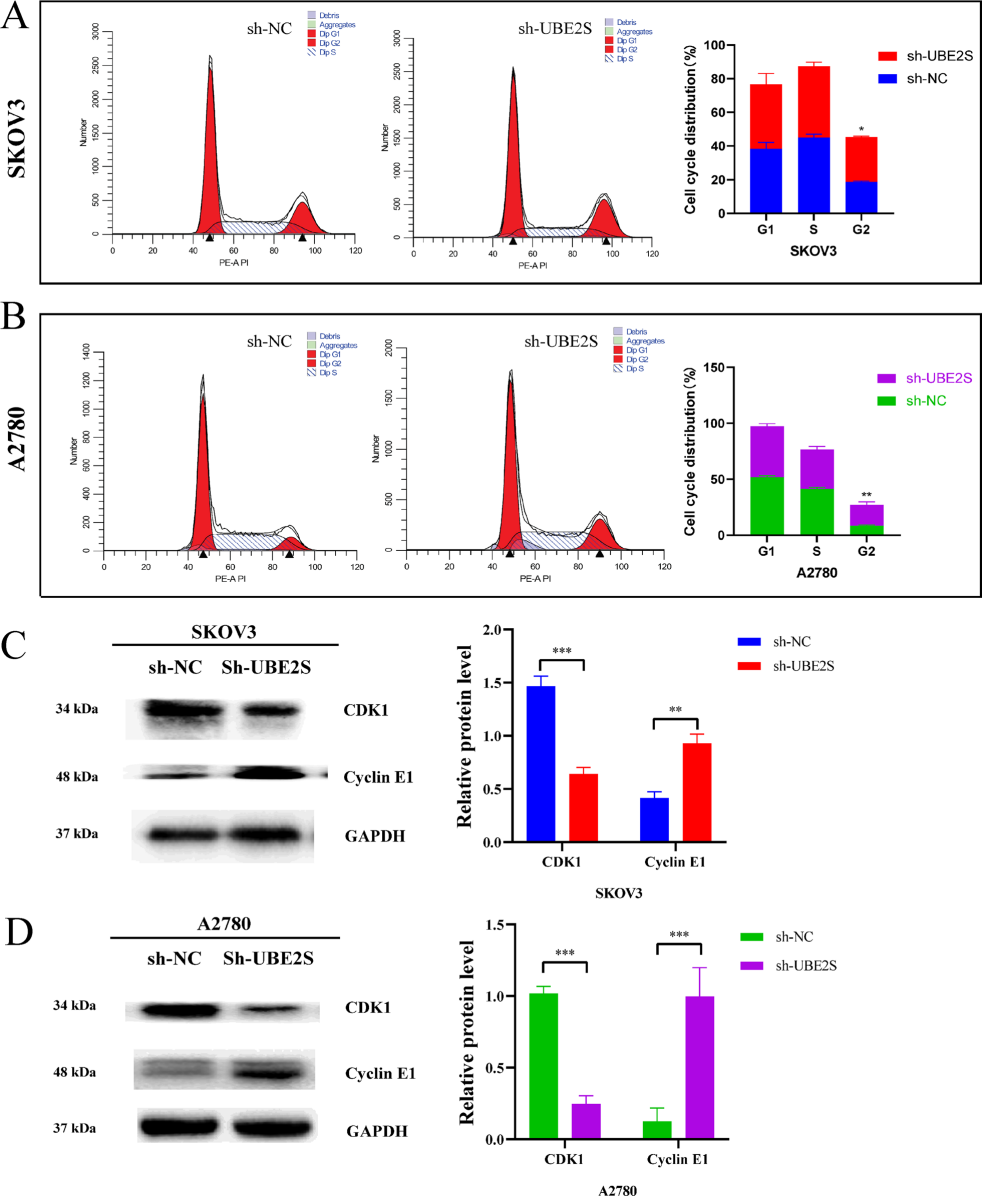
The impact of UBE2S gene knockdown on cell cycle. **A** Cell cycle-related flow cytometry results and statistics of SKOV3 cells in sh-NC group and sh-UBE2S group. **B** Cell cycle-related flow cytometry results and statistics of A2780 cells in sh-NC group and sh-UBE2S group. **C** Western blot results and statistics of cell cycle-related proteins of SKOV3 cells. **D** Western blot results and statistics of cell cycle-related proteins of A2780 cells

First, the results of the KEGG analysis showed that UBE2S may participate in the cell cycle regulation of OV cells, which is worthy of further study (Additional file 5: Fig. S2). Therefore, flow cytometry was performed to investigate the cell cycle in OV cells after UBE2S knockdown. The results of cell cycle-related flow cytometry indicated that, in A2780 and SKOV3 cell lines, the percentage of the G2 phase cell in the sh-UBE2S group was higher than the percentage in the sh-NC group (Fig. 10A, B). This result suggested that knockdown of UBE2S caused the cell cycle of OV cells to arrest in G2 phase.

Results of western blotting of cell cycle-related proteins indicated that, in A2780 and SKOV3 cell lines, the expression of CDK1 characteristic protein in the sh-NC group were higher than the expression in the sh-UBE2S group, and the expression of Cyclin E1 characteristic protein in the sh-NC group were lower than the expression in the sh-UBE2S group in OV cell lines (Fig. 10C and D). Cyclin A/B-CDK1 complex promotes the transition of cell cycle from G2 phase to M phase. The decrease of CDK1 characteristic protein in sh-UBE2S group may inhibit the transition of cell cycle from G2 phase to M phase. The Cyclin E1-CDK2 complex promotes the transition of the cell cycle from G1 to S and G2 phases. The increase of Cyclin E1 characteristic protein in sh-UBE2S group may promote the transition of cell cycle from G1 phase to S phase and G2 phase. According to the above results, it could be seen that the UBE2S gene knockdown might stagnate the cell cycle of OV cells in G2 phase by inhibiting the protein CDK1 and promoting the expression of Cyclin E1.

**Fig. 11:**
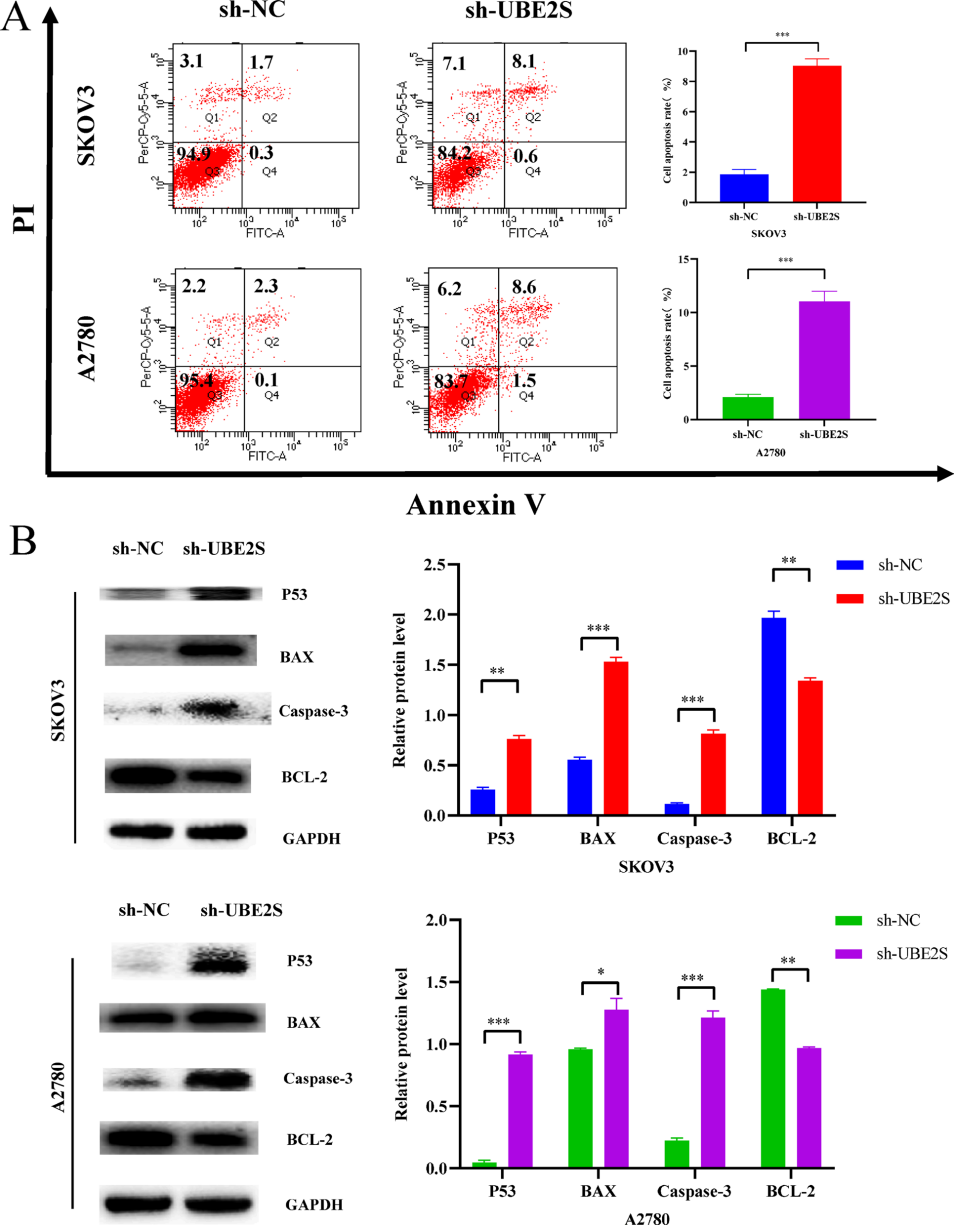
Effects of UBE2S gene knockdown on apoptosis. **A** Flow cytometry results and statistics related to apoptosis of ovarian cancer cells in SKOV3 cells and A2780 cells. **B** Western blot results and statistics of apoptosis-related proteins in SKOV3 cells and A2780 cells

**Fig. 12:**
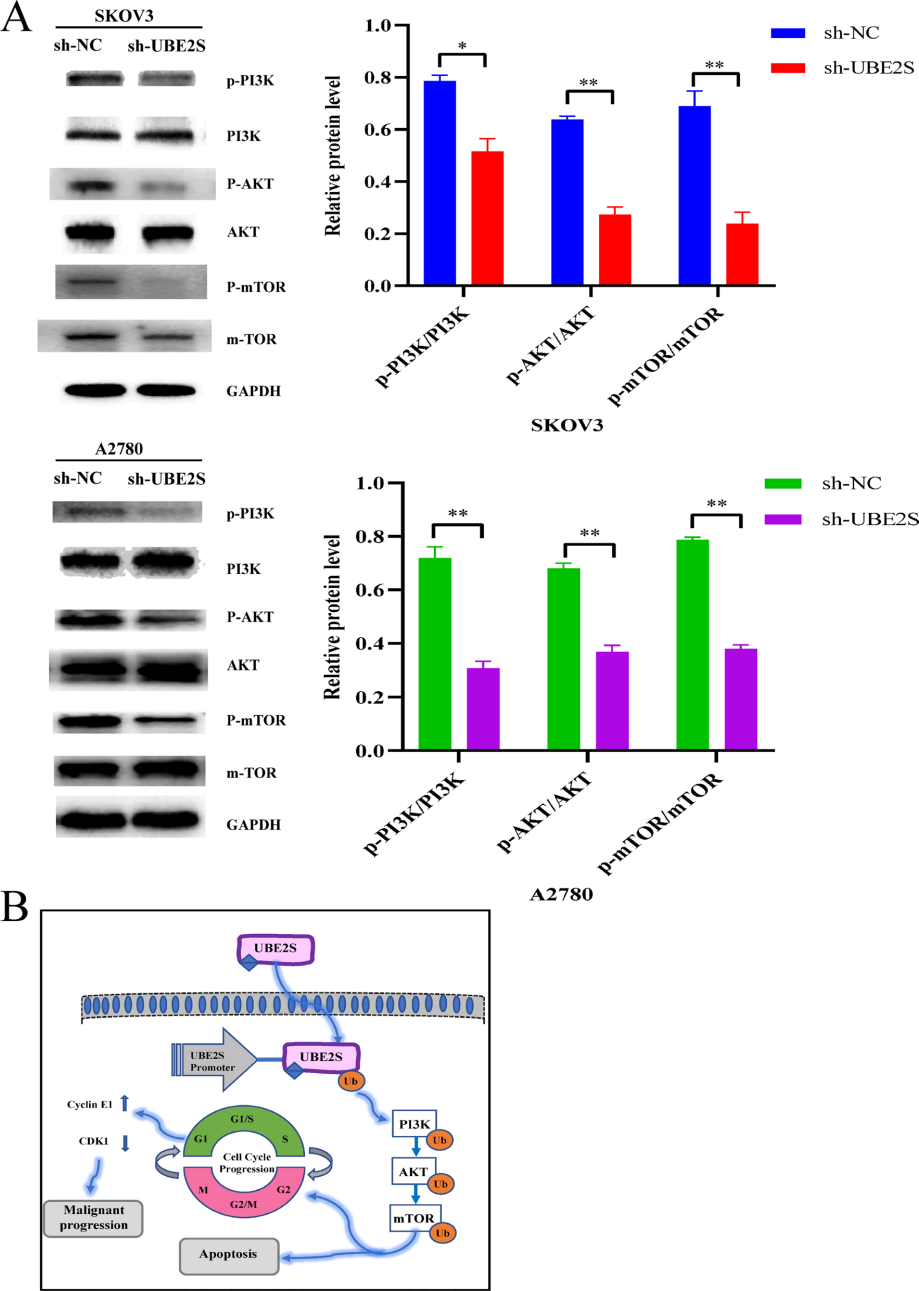
Western blotting results of PI3K/AKT/mTOR pathway related to UBE2S gene knockdown. **A** Western blotting results and statistics of p-PI3K/PI3K, p-AKT/AKT and p-mTOR/mTOR in SKOV3 and A2780 cell line. **B** The schematic diagram of the underlying mechanism of the UBE2S gene involved in regulating the malignant phenotype of OV based on this study

After that, Fig. 11 showed the results of apoptosis-related exploration. First of all, the apoptosis-related flow cytometry results in Fig. 11A showed that in SKOV3 and A2780 cell lines, the percentages of cells in apoptotic state (Q2 and Q4 regions) in the sh-UBE2S group were significantly higher than those in the sh-NC group (P < 0.05). Afterwards, the results of Western blotting of apoptosis proteins related to Fig. 11B showed that the relative expression levels of apoptosis positive regulators (P53, BAX and Caspase-3) were significantly higher in SKOV3 and A2780 cell lines compared with sh-NC group (P < 0.05), and the relative expression level of apoptosis negative regulator protein (BCL-2) was significantly lower than that in sh-NC group (P < 0.05). Therefore, the results of apoptosis-related flow cytometry and western blotting together suggested that the knockdown of UBE2S may promote the apoptosis of ovarian cancer cells by regulating the expression of apoptosis-specific proteins. Finally, considering that the PI3K/AKT/mTOR signaling pathway is commonly activated in ovarian cancer and plays an important role in the regulation of cell cycle and apoptosis, we attempted to explore the effect of UBE2S knockdown on the PI3K/AKT/mTOR signaling pathway. The Western blot results of Fig. 12A showed that the relative expression levels of PI3K/AKT/mTOR signaling pathway signature proteins (p-PI3K/PI3K, p-AKT/AKT and p-mTOR/mTOR) in SKOV3 and A2780 cell lines were significantly lower than that in the sh-NC group (P < 0.05), suggesting that the knockdown of UBE2S may inhibit the PI3K/AKT/mTOR signaling pathway. In summary, the results of the cell cycle, apoptosis and PI3K/AKT/mTOR signaling pathway research showed that the knockdown of UBE2S may affect the expression of cell cycle and apoptosis characteristic proteins by inhibiting the PI3K/AKT/mTOR signaling pathway. Thus, the cell cycle was blocked in the G2 phase, and at the same time, apoptosis was promoted, and finally the malignant biological behavior of ovarian cancer cells was inhibited. The underlying mechanisms were summarized and plotted in Fig. 12B.

## Discussion

Given the important role of UBE2S in several cancers, this study attempted to explore the expression level, clinical significance, and molecular mechanism of action of UBE2S in pan-gynecological cancers, especially in OV, through high-throughput data analysis and subsequent validation using clinical samples and in vitro experiments(Chandra et al. 2019; Ho et al. 2021; Stewart et al. 2019; Tang et al. 2021). The experimental flow chart is shown in Additional file 6: Fig. S3.

Massive amounts of genomic data deposited in public repositories as TCGA, GEPIA, GEO, and HPA have facilitated researchers to analyze large datasets and derive meaningful information (Barrett et al. 2013; Song et al. 2020; Tang et al. 2017; Wang et al. 2016). Our analysis revealed that UBE2S was significantly overexpressed in gynecological cancers (OV, UCEC, and CESC) at the pan-cancer level (Figs. 1, 2). In OV, the abnormally overexpression of UBE2S was closely related to a poorer prognosis of OV (Figs. 2C and 3C, D). To verify the reliability of the data analyzed from public databases, a complete set of in vitro experiments was performed on cell lines and clinical samples. The results of the experiments indicated that UBE2S was abnormally highly expressed in OV cell lines at the protein and mRNA levels (Fig. 3E, F). Analysis of the clinical samples confirmed the abnormally high expression of UBE2S in OV and its prognostic significance at the histological level (Figs. 4, 5). In conclusion, based on analysis of public datasets and clinical samples, the abnormally high expression and important clinical significance of UBE2S in OV were confirmed. The results confirmed that UBE2S is an oncogene with important prognostic significance in OV. Similarly, research that combined public data and in vitro experiments to verify each other was not uncommon, which also proved the scientific nature of this study from the side (Alnafakh et al. 2021; Heo et al. 2020; Neilsen et al. 2019).

It is generally accepted that oncogenes could affect the clinical prognosis of cancer patients by influencing the biological behavior of cancer cells (migration, invasion, and proliferation) (Leucci et al. 2016; Zhang et al. 2021a). Molecular biology experiments have been widely performed to explore the biological behavior of cancer cells (Henriques et al. 2021; Islam et al. 2021). After probing the abnormal expression of UBE2S in OV (origin) and its prognostic significance (outcome), the influence of UBE2S on the biological behavior of OV cells (intermediate process) was assessed through a complete set of cytological experiments. The results in Fig. 6 confirmed that the UBE2S gene knockdown efficiency was sufficient, which provided a basis for further research. Furthermore, the inhibitory effect of UBE2S knockdown on malignant biological processes, including proliferation and migration, of OV cells was confirmed (Figs. 7, 8, 9). Combining the results of the previous part of this study, we hypothesized that UBE2S, as a potential oncogene, might promote the malignant progression of OV and could lead to poor prognosis by promoting the malignant biological behavior of OV cells. Most importantly, many studies have reported that UBE2S may promote the malignant progression of hepatocellular carcinoma, non-small cell lung cancer, lung adenocarcinoma, and even endometrial cancer by promoting cell proliferation and other malignant biological processes, leading to poor prognosis (Lin et al. 2019b; Liu and Xu 2018; Pan et al. 2018; Qin et al. 2020). Therefore, the results and hypothesis of this study are supported by those of previous studies.

Oncogenes often affect the biological behavior of cells by participating in cell signaling pathways. In this regard, studies have shown that UBE2S promotes the development of hepatocellular carcinoma by inhibiting the p53 signaling pathway by enhancing the ubiquitination of p53 (Pan et al. 2018). In addition, other studies have shown that UBE2S could inhibit the tumor progression of endometrial cancer through the SOX6/β-Catenin signaling pathway (Lin et al. 2019a). However, whether UBE2S affects some cell signaling pathways in ovarian cancer still needs to be further explored in detail. Cell signaling pathways are complex and numerous, and often need to be predicted first. KEGG and GO analyses are widely performed to predict and screen cell signaling pathways and molecular mechanisms. In this study, the results of KEGG and GO analyses suggested that UBE2S might regulate the cell cycle (Additional file 5: Fig. S2). The results in Fig. 10 suggested that the knockdown of UBE2S may regulate the expression levels of cell cycle signature proteins (CDK1 and Cyclin E1) and then arrest the cell cycle of ovarian cancer cells in the G2 phase. The results in Fig. 11 suggested that the knockdown of UBE2S may enhance the expression levels of apoptosis characteristic proteins (P53, BAX and Caspase-3) and then promote the apoptosis of ovarian cancer cells. The results in Fig. 12 suggested that the knockdown of UBE2S may inhibit the expression levels of the characteristic proteins (p-PI3K/PI3K, p-AKT/AKT and p-mTOR/mTOR), thereby inhibiting ovarian PI3K/AKT/mTOR signaling pathway in ovarian cancer cells. Taking these results of this study together (Figs. 10, 11 and 12), it was reasonable to speculate that UBE2S may promote the malignant progression of ovarian cancer cells by promoting the PI3K/AKT/mTOR signaling pathway and thereby promoting cell cycle and inhibiting apoptosis. Studies have shown that UBE2S accelerates the cell cycle and promotes the development of hepatocellular carcinoma through the ubiquitination of p27 (Zhang et al. 2021b). In addition, studies have shown that knockdown of UBE2S promotes apoptosis, inhibits cell proliferation, and inhibits the mTOR signaling pathway in vitro and in vivo, thereby ultimately inhibiting the tumor progression of bladder cancer (Tang et al. 2021). These studies corroborate the rationality and scientificity of our speculation from the side.

Overall, this study showed the abnormally high expression of UBE2S at the pan-cancer level and gynecological cancers, especially OC. The overexpression of UBE2S was correlated with a poor prognosis. UBE2S was shown to promote the malignant biological behavior of OV cells. Furthermore, knockdown of UBE2S may inhibit the malignant progression of OV cells by inhibiting PI3K/AKT/mTOR to arrest cell cycle and promote apoptosis. These findings provide new avenues for the molecular therapy of OV. Therefore, this study not only comprehensively explored the expression level of UBE2S in OV, but also assessed the clinical prognostic significance and detailed role of UBE2S in OV at both transcriptomic and proteomic levels. However, this study has certain limitations. First, the experimental identification of the comprehensive molecular mechanism of action of UBE2S in ovarian cancer was not comprehensive. Moreover, we did not explore the detailed molecular mechanism of action of UBE2S in the context of post-translational modifications such as ubiquitination in OV cells, which might be the direction of further research.

## Conclusion

In summary, through multiple public data, clinical sample collection, and a series of in vitro experiments, UBE2S was found to be overexpressed in OV, possibly leading to poor prognosis. Furthermore, UBE2S might accelerate the cell cycle and inhibit apoptosis by promoting PI3K/AKT/mTOR and ultimately drive the malignant biological behavior of OV cells, which may provide new ideas for prognostic evaluation and molecular therapy of ovarian cancer.

## Supplementary Information

**Additional file 1: Table S1. **TCGA database and GTEx database sample summary

**Additional file 2. Table S2.** Expression level of UBE2S gene in pan-cancer.

**Additional file 3: Table S3. **Summary of Antibody Information.

**Additional file 4: Figure S1.** The expression, gene mutation and meta-analysis of UBE2S at the pan-cancer level in the ONCOMINE database. (A) The expression of UBE2S at the pan-cancer level. UBE2S was highly expressed in OV. The red square represented high expression; the blue square represented low expression; the shade of color represented the degree of high expression or low expression. (B) Genetic alteration and copy number variation of UBE2S in OV. The percentages of each situation were clarified. CNV (Copy number variation); Hete Amp (Heterogeneous amplification); Homo Amp (Homologous amplification); Hete Del (Heterologous deletion); Homo Del (Homologous deletion). (C) A meta-analysis of UBE2S expression levels in the data sets of four different studies.

**Additional file 5: Figure S2. **Prediction of the underlying mechanism of UBE2S in OV. (A) The schematic diagram of enrichment analysis of KEGG pathway related to UBE2S gene, which represented the molecular mechanism or signal pathway that the UBE2S gene may participate in the regulation. The larger the circle, the closer the relationship. (B) Annotation and statistical significance of enrichment analysis of KEGG pathway related to UBE2S gene. (C) The schematic diagram of the GO analysis related to the UBE2S gene, representing the molecular mechanism by which the UBE2S gene may be involved in regulation. The longer the arc, the closer the relationship. (D) Annotation and statistical significance of GO analysis related to UBE2S gene. BP, Biological process; CC, Cell components; MF, Molecular function.

**Additional file 6: Figure S3.** Schematic diagram of the process.

## Data Availability

The data can be obtained through the email under reasonable request: 1427@hrbmu.edu.cn.

## Abbreviations

OV: Ovarian cancer
CESC: Endocervical adenocarcinoma
UCEC: Uterine corpus endometrial carcinoma
UBE2S: Ubiquitin-conjugating enzyme E2S
UBE1: Ubiquitin-like modifier activating enzyme 1
UBE3: Ubiquitin-protein ligase E3
TCGA: The Cancer Genome Atlas
GEPIA: The Gene Expression Profiling Interactive Analysis
GEO: Gene Expression Omnibus
IHC: Immunohistochemistry
