# Supplementary material for: UBE2S promotes the development of ovarian cancer by promoting PI3K/AKT/mTOR signaling pathway to regulate cell cycle and apoptosis
Source: Mol Med. 2022 Jun 3;28:62. doi: 10.1186/s10020-022-00489-2 (PMC9166599; doi:10.1186/s10020-022-00489-2)
Supplement: Supplementary file 1 — Additional file 1: Table S1. TCGA database and GTEx database sample summary [file 10020_2022_489_MOESM1_ESM.docx]

**Table S1. TCGA database and GTEx database sample summary**

| **Cancer Type** | **Full name** | **Tumor** | **Normal**（TCGA） | **Normal**（GTEx） |
| --- | --- | --- | --- | --- |
| ACC | Adrenocortical carcinoma | 77 |  | 128 |
| BLCA | Bladder Urothelial Carcinoma | 407 | 19 | 9 |
| BRCA | Breast invasive carcinoma | 1099 | 113 | 179 |
| CESC | Cervical squamous cell and endocervical adenocarcinoma | 306 | 3 | 10 |
| CHOL | Cholangiocarcinoma | 36 | 9 |  |
| COAD | Colon adenocarcinoma | 290 | 41 | 308 |
| DLBC | Lymphoid Neoplasm Diffuse Large B-cell Lymphoma | 47 |  | 444 |
| ESCA | Esophageal carcinoma | 182 | 13 | 653 |
| GBM | Glioblastoma multiforme | 166 | 5 | 1152 |
| HNSC | Head and Neck squamous cell carcinoma | 502 | 44 |  |
| KICH | Kidney Chromophobe | 66 | 25 | 28 |
| KIRC | Kidney renal clear cell carcinoma | 531 | 72 | 28 |
| KIRP | Kidney renal papillary cell carcinoma | 289 | 32 | 28 |
| LAML | Acute Myeloid Leukemia | 173 |  | 70 |
| LGG | Brain Lower Grade Glioma | 523 |  | 1152 |
| LIHC | Liver hepatocellular carcinoma | 371 | 50 | 110 |
| LUAD | Lung adenocarcinoma | 515 | 59 | 288 |
| LUSC | Lung squamous cell carcinoma | 498 | 50 | 288 |
| MESO | Mesothelioma | 87 |  |  |
| OV | Ovarian serous cystadenocarcinoma | 427 |  | 88 |
| PAAD | Pancreatic adenocarcinoma | 179 | 4 | 167 |
| PCPG | Pheochromocytoma and Paraganglioma | 182 | 3 |  |
| PRAD | Prostate adenocarcinoma | 496 | 52 | 100 |
| READ | Rectum adenocarcinoma | 93 | 10 | 308 |
| SARC | Sarcoma | 262 | 2 |  |
| SKCM | Skin Cutaneous Melanoma | 469 | 1 | 812 |
| STAD | Stomach adenocarcinoma | 414 | 36 | 174 |
| TGCT | Testicular Germ Cell Tumors | 154 | 262 | 165 |
| THCA | Thyroid carcinoma | 512 | 59 | 279 |
| THYM | Thymoma | 119 | 2 | 444 |
| UCEC | Uterine Corpus Endometrial Carcinoma | 181 | 23 | 78 |
| UCS | Uterine Carcinosarcoma | 57 |  | 78 |
| UVM | Uveal Melanoma | 79 |  |  |
