# Supplementary material for: UBE2S promotes the development of ovarian cancer by promoting PI3K/AKT/mTOR signaling pathway to regulate cell cycle and apoptosis
Source: Mol Med. 2022 Jun 3;28:62. doi: 10.1186/s10020-022-00489-2 (PMC9166599; doi:10.1186/s10020-022-00489-2)
Supplement: Supplementary file 2 — Additional file 2. Table S2. Expression level of UBE2S gene in pan-cancer. [file 10020_2022_489_MOESM2_ESM.docx]

| **Tumor** |  | **number** | **minimum** | **maximum** | **Median** |
| --- | --- | --- | --- | --- | --- |
| ACC | Normal | 128 | 0 | 5.218 | 3.716 |
| ACC | Tumor | 77 | 2.029 | 8.401 | 5.688 |
| BLCA | Normal | 28 | 3.511 | 6.8 | 4.557 |
| BLCA | Tumor | 407 | 3.296 | 9.623 | 6.382 |
| BRCA | Normal | 292 | 1.888 | 4.959 | 3.423 |
| BRCA | Tumor | 1099 | 2.534 | 8.543 | 5.503 |
| CESC | Normal | 13 | 3.036 | 5.026 | 3.896 |
| CESC | Tumor | 306 | 4.374 | 8.871 | 6.723 |
| CHOL | Normal | 9 | 1.465 | 2.776 | 2.147 |
| CHOL | Tumor | 36 | 3.602 | 6.837 | 4.862 |
| COAD | Normal | 349 | 0 | 6.522 | 3.919 |
| COAD | Tumor | 290 | 2.59 | 8.098 | 6.246 |
| DLBC | Normal | 444 | 0.678 | 7.472 | 3.411 |
| DLBC | Tumor | 47 | 5.435 | 8.436 | 6.856 |
| ESCA | Normal | 666 | 0 | 6.561 | 4.17 |
| ESCA | Tumor | 182 | 3.792 | 7.926 | 6.146 |
| GBM | Normal | 1157 | 0 | 8.323 | 4.387 |
| GBM | Tumor | 166 | 3.856 | 8.806 | 6.232 |
| HNSC | Normal | 44 | 1.17 | 5.543 | 4.734 |
| HNSC | Tumor | 520 | 3.61 | 8.623 | 6.49 |
| KICH | Normal | 53 | 0 | 5.348 | 3.814 |
| KICH | Tumor | 66 | 2.373 | 6.52 | 4.46 |
| KIRC | Normal | 100 | 0 | 5.111 | 3.433 |
| KIRC | Tumor | 531 | 0.632 | 7.424 | 3.761 |
| KIRP | Normal | 60 | 0 | 5.03 | 3.528 |
| KIRP | Tumor | 289 | 2.208 | 6.466 | 3.882 |
| LAML | Normal | 70 | 6.733 | 8.131 | 7.463 |
| LAML | Tumor | 173 | 3.36 | 6.353 | 4.843 |
| LGG | Normal | 1152 | 0 | 8.323 | 4.384 |
| LGG | Tumor | 523 | 3.246 | 8.417 | 5.063 |
| LIHC | Normal | 160 | 0.705 | 5.637 | 2.276 |
| LIHC | Tumor | 371 | 1.77 | 8.027 | 4.356 |
| LUAD | Normal | 347 | 0 | 5.453 | 3.885 |
| LUAD | Tumor | 515 | 1.844 | 8.821 | 5.011 |
| LUSC | Normal | 338 | 0 | 5.453 | 3.952 |
| LUSC | Tumor | 498 | 3.483 | 10.1 | 6.717 |
| MESO | Tumor | 87 | 3.503 | 7.263 | 5.323 |
| OV | Normal | 88 | 2.356 | 5.062 | 3.645 |
| OV | Tumor | 427 | 0 | 8.527 | 6.242 |
| PAAD | Normal | 171 | 0 | 4.908 | 1.748 |
| PAAD | Tumor | 179 | 1.753 | 7.749 | 4.967 |
| PCPG | Normal | 3 | 3.789 | 5.044 | 4.36 |
| PCPG | Tumor | 182 | 4.037 | 7.265 | 5.714 |
| PRAD | Normal | 152 | 2.755 | 5.615 | 4.031 |
| PRAD | Tumor | 496 | 2.92 | 7.336 | 4.685 |
| READ | Normal | 318 | 0 | 6.522 | 3.888 |
| READ | Tumor | 93 | 3.531 | 7.592 | 6.057 |
| SARC | Normal | 2 | 3.608 | 4.818 | 4.213 |
| SARC | Tumor | 262 | 3.603 | 9.091 | 6.287 |
| SKCM | Normal | 813 | 1.774 | 8.212 | 3.545 |
| SKCM | Tumor | 469 | 3.417 | 8.628 | 6.5 |
| STAD | Normal | 210 | 0 | 5.679 | 3.503 |
| STAD | Tumor | 414 | 2.618 | 8.716 | 5.84 |
| TGCT | Normal | 165 | 3.109 | 8.166 | 7.562 |
| TGCT | Tumor | 154 | 3.708 | 8.448 | 7.23 |
| THCA | Normal | 338 | 0 | 5.809 | 3.54 |
| THCA | Tumor | 512 | 2.524 | 7.509 | 3.923 |
| THYM | Normal | 446 | 0.678 | 7.472 | 3.415 |
| THYM | Tumor | 119 | 3.175 | 7.689 | 6.397 |
| UCEC | Normal | 101 | 2.616 | 5.805 | 3.952 |
| UCEC | Tumor | 181 | 2.631 | 8.589 | 6.189 |
| UCS | Normal | 78 | 2.616 | 5.619 | 3.82 |
| UCS | Tumor | 57 | 5.659 | 9.894 | 6.948 |
| UVM | Tumor | 79 | 3.165 | 5.932 | 4.78 |

**Table S2.Expression level of UBE2S gene in pan-cancer**
