# Supplementary material for: UBE2S promotes the development of ovarian cancer by promoting PI3K/AKT/mTOR signaling pathway to regulate cell cycle and apoptosis
Source: Mol Med. 2022 Jun 3;28:62. doi: 10.1186/s10020-022-00489-2 (PMC9166599; doi:10.1186/s10020-022-00489-2)
Supplement: Supplementary file 3 — Additional file 3: Table S3. Summary of Antibody Information. [file 10020_2022_489_MOESM3_ESM.docx]

| Antibody Name | Manufacturer name |
| --- | --- |
| UBE2S | Absin |
| GAPDH | Affinity Biosciences |
| CDK1 | Bioworld |
| Cyclin E1 | Bioworld |
| PI3K  p-PI3K | Affinity Biosciences  Affinity Biosciences |
| AKT | Cell Signaling Technology |
| p-AKT | Cell Signaling Technology |
| mTOR | Cell Signaling Technology |
| p-mTOR | Cell Signaling Technology |
| BCL-2 | Absin |
| P53 | Absin |
| Bax | Cell Signaling Technology |

**Table S3. Summary of Antibody Information**
