# Supplementary figures and images for: UBE2S promotes the development of ovarian cancer by promoting PI3K/AKT/mTOR signaling pathway to regulate cell cycle and apoptosis
Source: Mol Med. 2022 Jun 3;28:62. doi: 10.1186/s10020-022-00489-2 (PMC9166599; doi:10.1186/s10020-022-00489-2)

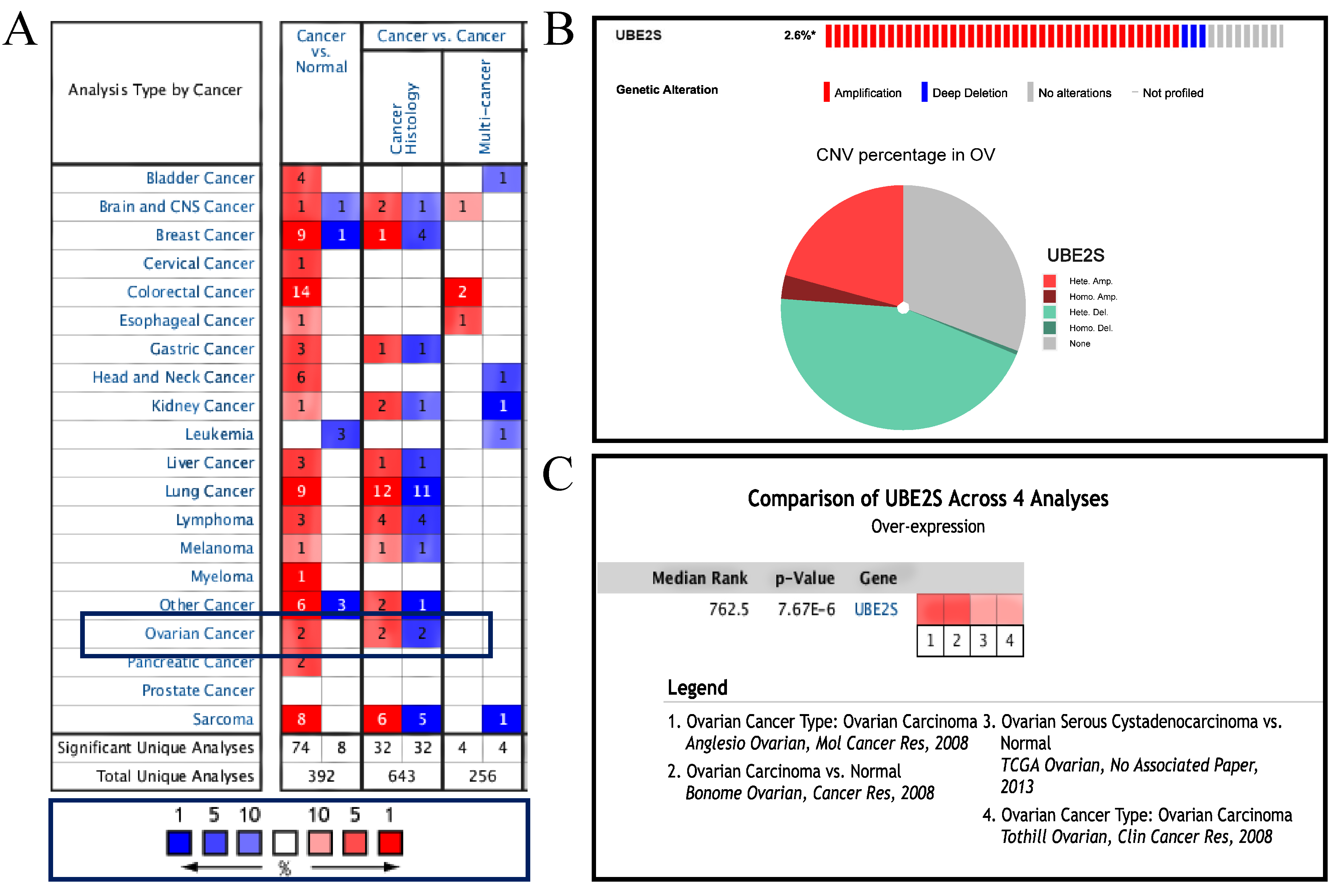

Supplement: Supplementary file 4 — Additional file 4: Figure S1. The expression, gene mutation and meta-analysis of UBE2S at the pan-cancer level in the ONCOMINE database. (A) The expression of UBE2S at the pan-cancer level. UBE2S was highly expressed in OV. The red square represented high expression; the blue square represented low expression; the shade of color represented the degree of high expression or low expression. (B) Genetic alteration and copy number variation of UBE2S in OV. The percentages of each situation were clarified. CNV (Copy number variation); Hete Amp (Heterogeneous amplification); Homo Amp (Homologous amplification); Hete Del (Heterologous deletion); Homo Del (Homologous deletion). (C) A meta-analysis of UBE2S expression levels in the data sets of four different studies. [file 10020_2022_489_MOESM4_ESM.tif]

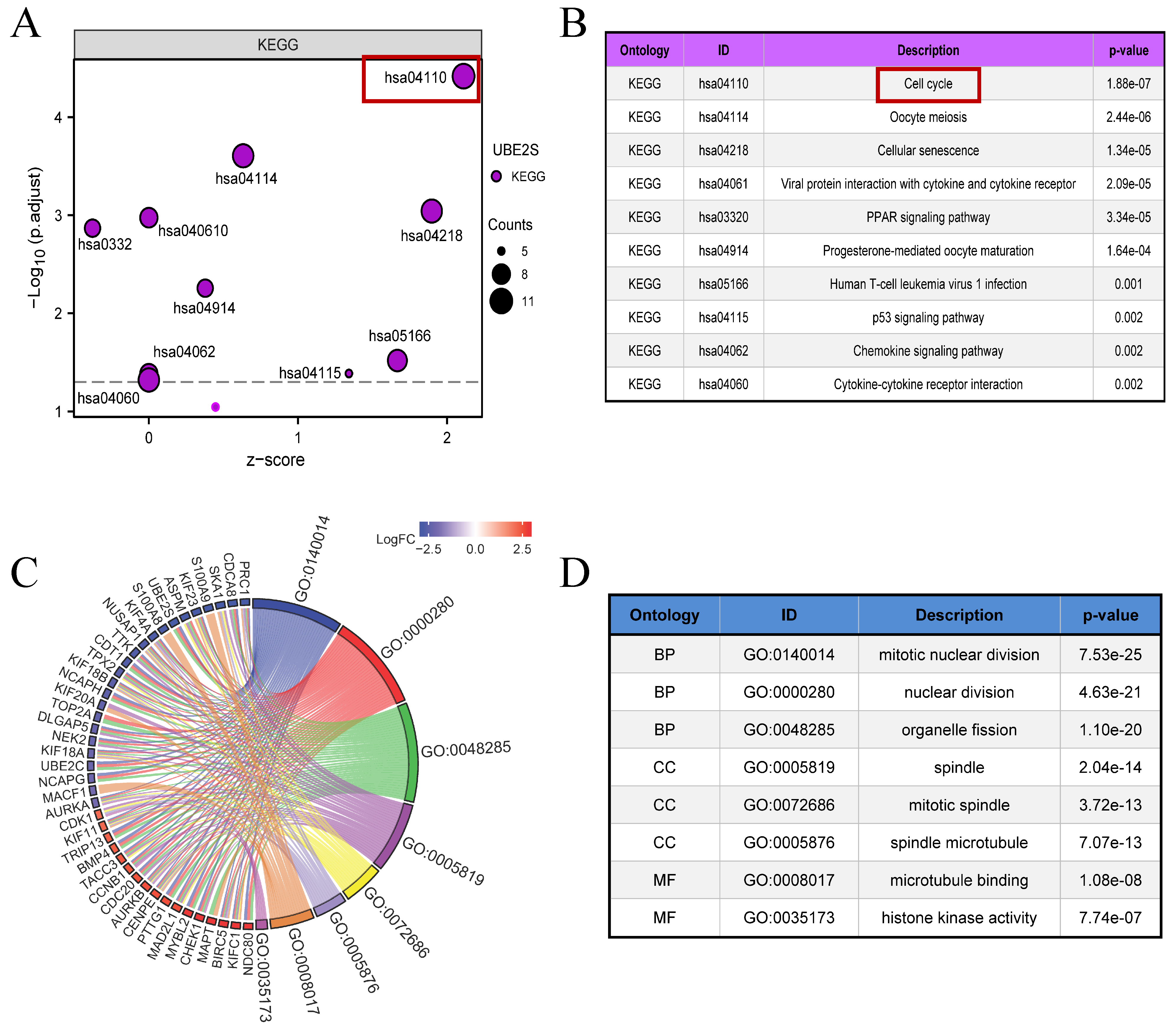

Supplement: Supplementary file 5 — Additional file 5: Figure S2. Prediction of the underlying mechanism of UBE2S in OV. (A) The schematic diagram of enrichment analysis of KEGG pathway related to UBE2S gene, which represented the molecular mechanism or signal pathway that the UBE2S gene may participate in the regulation. The larger the circle, the closer the relationship. (B) Annotation and statistical significance of enrichment analysis of KEGG pathway related to UBE2S gene. (C) The schematic diagram of the GO analysis related to the UBE2S gene, representing the molecular mechanism by which the UBE2S gene may be involved in regulation. The longer the arc, the closer the relationship. (D) Annotation and statistical significance of GO analysis related to UBE2S gene. BP, Biological process; CC, Cell components; MF, Molecular function. [file 10020_2022_489_MOESM5_ESM.tif]

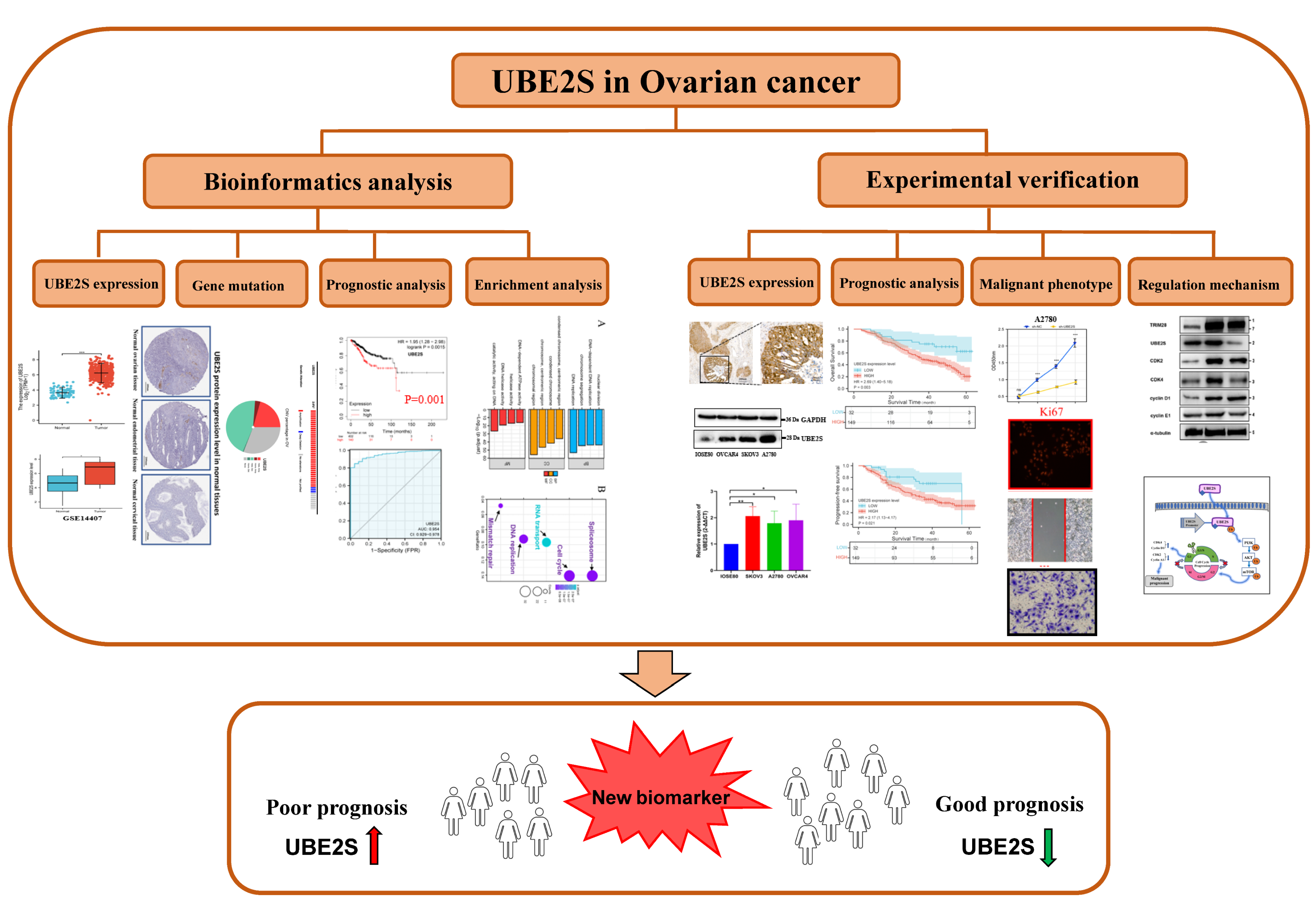

Supplement: Supplementary file 6 — Additional file 6: Figure S3. Schematic diagram of the process. [file 10020_2022_489_MOESM6_ESM.tif]
